# Evolutionary unpredictability in cancer model systems

**DOI:** 10.1038/s41598-025-07407-6

**Published:** 2025-06-27

**Authors:** Subhayan Chattopadhyay, Jenny Karlsson, Michele Ferro, Adriana Mañas, Ryu Kanzaki, Elina Fredlund, Andrew J. Murphy, Christopher L. Morton, Natalie Andersson, Mary A. Woolard, Karin Hansson, Katarzyna Radke, Andrew M. Davidhoff, Sofie Mohlin, Kristian Pietras, Daniel Bexell, David Gisselsson

**Affiliations:** 1 Division of Clinical Genetics, Department of Laboratory Medicine, Lund University, Lund, Sweden; 2 Division of Translational Cancer Research, Department of Laboratory Medicine, Lund University, Lund, Sweden; 3 Translational Research in Pediatric Oncology and Cell Therapy Group, IdiPAZ, University Hospital La Paz, Madrid, Spain; 4 Pediatric Oncohematology, Pediatric Oncohematology Clinical Unit, National Cancer Research Center (CNIO), Madrid, Spain; 5 Division of Pediatrics, Clinical Sciences, Translational Cancer Research, Lund University, Lund, Sweden; 6 Lund Stem Cell Center, Lund University, Lund, Sweden; 7 Lund University Cancer Center, Lund University, Lund, Sweden; 8 Department of Surgery, St. Jude Children’s Research Hospital, Memphis, USA; 9 Clinical Genetics, Pathology and Molecular Diagnostics, Office of Medical Services, Region Skåne, Sweden

**Keywords:** Cancer, Cancer genetics, Cancer models

## Abstract

Despite the advent of advanced molecular prognostic tools, it is still difficult to predict the course of disease for cancer patients at the individual level. This lack of predictability is also reflected in many experimental cancer model systems, begging the question of whether certain biological aspects of cancer (eg. growth, evolution etc.) can ever be anticipated or if there remains an inherent unpredictability to cancer, similar to other complex biological systems. We demonstrate by a combination of agent-based mathematical modelling, analysis of patient-derived xenograft model systems from multiple cancer types, and in-vitro culture that certain conditions increase stochasticity of the clonal landscape of cancer growth. Our findings indicate that under those conditions, the cancer genome may behave as a complex dynamic system, making its long-term evolution inherently unpredictable.

## Introduction

In 1993, Donald S. Coffey hypothesized that cancer is an ‘abrupt’ and ‘emergent’ phenomenon caused by the transformation of the cell proliferation machinery from an ordered to a disordered, albeit self-organizing state^1^. The prevalent somatic mutation theory stating that near-random DNA lesions combined with disease-specific selection cause cancer still indicates a failure to grasp Coffey’s vision of using the self-organizing features of cancer to identify unifying aspects across malignancies^2–4^. With the advent of precision cancer medicine, major efforts are now being made to characterize the broad spectrum of genetic variation across individual tumors. This has resulted in a fine-tuned associative prognostication of many neoplasms and in identification of treatment targets based on molecular signatures. However, in many cases, the remaining threat of a relapse at an unknown time point, often manifesting as treatment-resistant aggressive disease, reminds us that cancer shares features of resilience with many other self-organizing systems. Cancer’s relapse mechanisms have been thoroughly studied through the lens of genetic diversity, elucidating how tumors evolve along different evolutionary trajectories^5^ and how resistant clones often appear due to excessive evolutionary branching early in disease, leading to dormant tumor cell populations with a potential to clonally sweep at later stages^6,7^. Despite the massive amount of data thus accumulated on the molecular routes of relapse, it remains an essentially unpredictable phenomenon. However, as inferred by Coffey decades earlier, cancer is not a purely stochastic phenomenon. Instead, it results from runaway tissue homeostasis, transitioning into a complex, dynamic, and adaptive system. This notion begs the question whether we can use generic knowledge from other complex systems to better understand tumorigenesis.

The generalized logistic model (with the Gompertz curve being a special case) is a commonly used construct for simulating spatially constricted (bounded) growth of species/cell populations, and it is often used to emulate solid tumor growth^8–10^. Several comparative studies analyzing in-vivo tumor growth have established the suitability of logistic functions in tumor growth estimation^10,11^. Additionally, logistic functions have been used to illustrate clonal selection and genetic drift in solid tumors in silico^12^. Simulations have shown that the evolutionary trajectories of cancers are highly dependent on how cell populations grow and how they interact with the stromal boundary^13^. Recently it was shown that radiological estimates from merely three distinct time-points can reliably predict the growth dynamics (based on logistic growth) of some solid tumors^14^. However, whether the logistic function as a mechanism for tumor growth can reliably predict emergent clonal geographies in tumorigenesis remains yet to be explored. Here, we probe this question with a focus on the predictability of mutational landscapes.

## Results

**Fig. 1:**
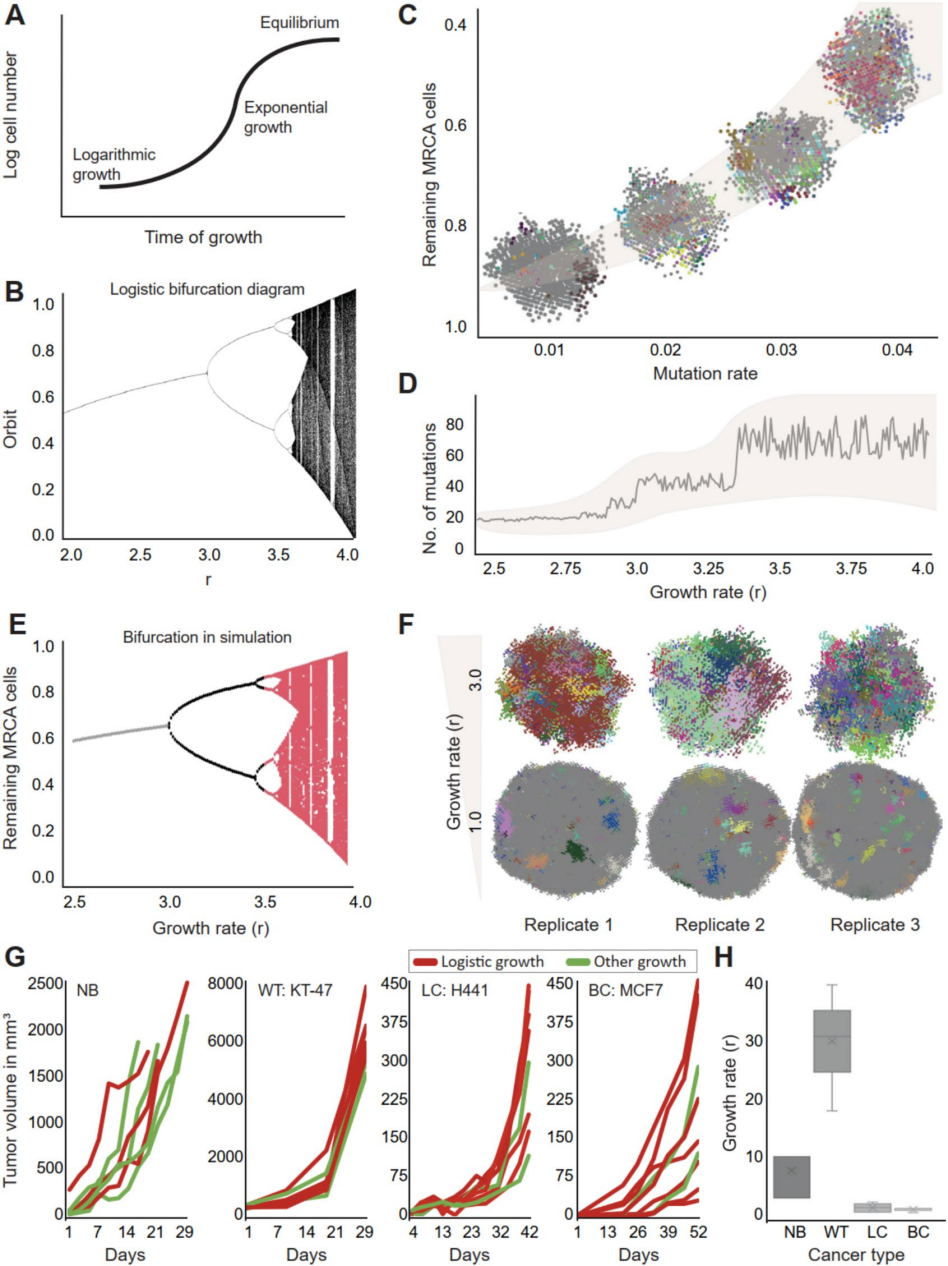
Simulation of tumor evolution and growth dynamics. (**A**) Interpretation of the classical shape of a logistic growth curve. (**B**) Classical orbit diagram of logistic curve (logistic map) 16. (**C**) Change in percentage of remaining MRCA (most recent common ancestors) cells was plotted against increasing mutation rate. Examples were overlayed at the estimated mean calculated with 100 simulation runs. Confidence interval with shaded overlay depicts 3σ limit. Please note the reversed axes. (**D**) Average number of total distinct mutations at the end of simulation is plotted against growth rates (r). All estimates are calculated over 100 separate runs. As the logistic map experiences chaotic fluctuations starting at r = 3.0, the x-axis is terminated at 4.0 with adequate variability depicted in the range (confidence interval depicts 3σ limit). (**E**) Cell growth rate was plotted against percentage of remaining ancestors at the end of simulations, grey section indicates r < 3.0, calculated by taking average; black section indicates 3.0 < r < 3.5, calculated by cluster centroids; red section indicates r > 3.5 where all estimates are plotted. (F) Three replicates of simulation are shown for growth rates (r) 1.0 and 3.0 to draw attention to the fact that higher ‘r’ results in markedly different mutational pattern compared to that in lower ‘r’. The colors in each simulation indicate a cluster of clonal cells. (**G**) Tumor growth data in mouse models are depicted; red: logistic growth and green: did not fit to logistic function. NB: neuroblastoma 21, WT: Wilms tumor (KT-47 sample, Murphy et. al.); LC: lung cancer (H441 cell line); BC: breast cancer (MCF7 cell line). (**H**) Box plots of growth rates of data shown in (1-G) plotted across cancer types. ‘X’ signifies the mean.

In his seminal work on chaotic oscillations in dynamic systems, James A. Yorke showed that a self-repeating process, with a periodicity of three or more, experiences chaotic fluctuations. This was exemplified by the logistic function^15^, which is a sigmoidal curve with a periodically oscillating slope (Fig. 1A). Robert May further demonstrated how chaotic fluctuations could appear as a function of logistic growth in a bifurcation diagram^16^ (Fig. 1B), presenting how a function can assume more than one value near its asymptotes (orbits).

To investigate the potential for non-linear variations in the clonal composition of proliferating cancer cells, we conducted computational modeling predicated on the hypothesis that neoplasms, before the onset of the latest clonal expansion, originate from a genetically homogeneous cell population, hereinafter designated as ancestor cells. These ancestor cells underwent clonal expansion adhering to certain parameters (i.e., a growth rate governed by cell division and death rates, the rate of acquiring mutations at each cell division, and the probability of a mutation to be a driver mutation), which were set at initiation and remained unchanged, purely for the sake of simplicity. The selective advantages provided by a random mutation were sourced from the deleteriousness scores provided by the COSMIC database (detailed in Methods)^17^ and the maximum number of cells at the end of the simulation was fixed. To monitor clonal landscapes at the end of simulations, we focused on how the cells underwent genetic diversification due to variations in simulation parameters, making them genetically distant progenies of the ancestors. All the cells at the end of each simulation were clustered according to the number of acquired mutations, and the least mutated cluster was considered to represent the most recent common ancestors. With this, we arrive at a simple measure of the percentage of ancestors remaining at the end of a simulation run. We evaluated the change in the percentage of the remaining ancestors against varying initial conditions to observe possible indications of dynamic oscillation.

With each simulation cycle, mutations were drawn at random from a set of known somatic mutations (see Methods). Using fixed initial conditions, we evaluated whether genetic diversification remained predictable, given certain mutation parameters. Keeping the growth rate unchanged, we first varied the probability of acquiring a mutation at each cell division between 0.01 and 0.04 (Fig. 1C)^12^. Growth rates were affected by mutation rate, mutation type and mutation accumulation all of which conferred changes in cellular fitness. We also observed a nonlinear monotonically decreasing relationship between the percentage of ancestors and an increasing mutation rate. The fraction of the remaining ancestors at the end of the simulation varied on average between 92% (low mutation rate) and 36% (high mutation rate, calculated over 100 simulation runs). However, the relationship between the median number of mutations and growth rate was less straightforward. Mutation acquisition seemed to undergo a punctuated step-like increment (Fig. 1D). Clear jumps in median mutation accumulation were observed near logistic growth rates (r) 3.0 and 3.4, indicating rapid changes in the distribution of mutation accumulation at certain growth rates. Indeed, the jumps in mutation accumulation corresponded to the classic bifurcation diagram of the logistic function (Fig. 1B)^16^, when plotting the percentage of the remaining ancestor against the growth rate (Fig. 1E). It’s crucial to emphasize that these metrics aren’t interchangeably interpreted and the stochasticity or lack thereof in the observed MRCA proportion depends on initial conditions of the model parameter. A faster growth rate resulted in a markedly heterogeneous mutational landscape compared to a slower growth rate (Fig. 1F). The shape of the bifurcation diagram thus describes unpredictable behavior, i.e. a one-to-many solution of the logistic map at the asymptotes, as the growth constant increases over a certain threshold. In our simulations, this was observed at a growth rate above 3.0. This led us to conclude that unpredictable growth (at least in this case) is a biologically emergent feature that can occur in tumors following logistic growth above a certain rate.

Talkington and Durett evaluated in vivo growth characteristics of several cancer cell lines and found that numerous cell lines at least partly resemble logistic (specifically, Gompertzian) growth^11^. Some of the earliest investigations on in vitro growth experiments have also pointed towards a similar pattern^18,19^. However, the extent to which fast logistic growth occurs in tumors in vivo remains unclear.

To evaluate whether fast-growing tumors with a potentially unpredictable clonal evolution exist, we assessed how often logistic growth is observed across in vivo model systems in situations simulating relapse by implantation of a limited tumor population. Pediatric cancers are well known to have much higher growth rates than adult cancers. Neuroblastoma (NB) and Wilms tumor (WT) are two of the most common solid pediatric tumors, and high-risk NB and WT are notorious for their rapid growth. We estimated growth rates from previously published data on untreated patient-derived xenografts (PDX) derived from NB and WT^5,20–23^. For comparison with adult tumors (i.e., slow growers)^24^, we also evaluated uninhibited growth of several lung and breast cancer cell lines. Strikingly, 43% of the evaluated NB PDXs abided to logistic growth (73% of growth rates were more than 3.0, with a median of 10.0), considerably above the bifurcation limit of 3.0 (Supplementary Tables 2,3,5), whereas 75% of the WT PDXs showed logistic growth (all growth rates over 3.0) with a median growth rate of 31.0 (Fig. 1G,H; Supplementary Table 6). In PDXs from the H441 lung cancer and MCF7 breast cancer cell lines, 71% and 78%, respectively, experienced logistic growth, but none over 3.0 (Supplementary Tables 7,8). The median growth rates were only 1.13 and 0.9, respectively, far below that for chaotic fluctuations. In addition to patient-derived models, we also evaluated the growth of two lineages of the NB SK-N-BE(2)C cell line (referred as BE cell line hereinafter) in vitro, with median growth rates of 5.0 and 4.0, respectively (Supplementary Table 4). When combined, the growth rates from all NB replicates had a median of 6.0, and those from WT replicates a median of 24.0. All but one breast cancer replicate that adhered to logistic growth had growth rates below 3.0, with a median of 0.99 and that for lung cancer replicates was 0.68, none reaching 3.0 (Supplementary Table 9).

The implications of the present study are limited to tumors that demonstrated a logistic growth curve; however, not all tumors did so. This is possibly due to different inherent growth characteristics, as the absolute volume of the tumors varies substantially between pediatric and adult tumors. By week four of observation, the average volume for the NB replicates exceeded 2000 mm^3^. In the same time frame, adult tumors grew only about 400 mm^3^ except for one replicate (m3) of the MDA-MB-231 breast cancer cell line (growth rate 3.6). The WT PDXs at implantation had a volume of at least 200 mm^3^ with an average size of 308 mm^3^ which grew to 3800 mm^3^ on average by week 3. Notably, NB PDXs underwent orthotopic implantation initially and were then re-engrafted heterotopically^23^ whereas the WTs were heterotopic. Often, the reason that a curve failed to reasonably fit a logistic function was due to abrupt changes in the growth pattern (measurement artifacts, etc.), which is not unexpected. Nevertheless, the presence of any systematic artifact responsible for the low growth rates in the adult tumors was ruled out, as the experiments yielded growth rate within-variances of 0.023 and 0.012.

To investigate experimentally the effects of varying growth rates on acquisition of mutations and subsequent shift in clonality, we then assessed growth dynamics of the BE neuroblastoma cell line. Standard experimental methods fall short in studying evolutionary changes in vitro due to their restriction to small populations, failing to mimic the extensive intra-tumor heterogeneity observed in most human cancers^25^. Current techniques for examining rapid genomic changes in cell culture systems involve prolonged exposure of cells to repeated bottlenecks, typically taking six months to a year^26^. Mutation rates in cancer per base per cell division vary approximately between 10^−8^ and 10^−7^, and even with T175 flasks, less than 10 passages (assuming 1:10 re-plating) are insufficient to have a single clonal mutant in the population. Therefore, small culture systems may be inadequate to observe a mutational drift reliably and may inadvertently drive the evolutionary dynamics towards a highly unpredictable and unstable mutational niche.

**Fig. 2:**
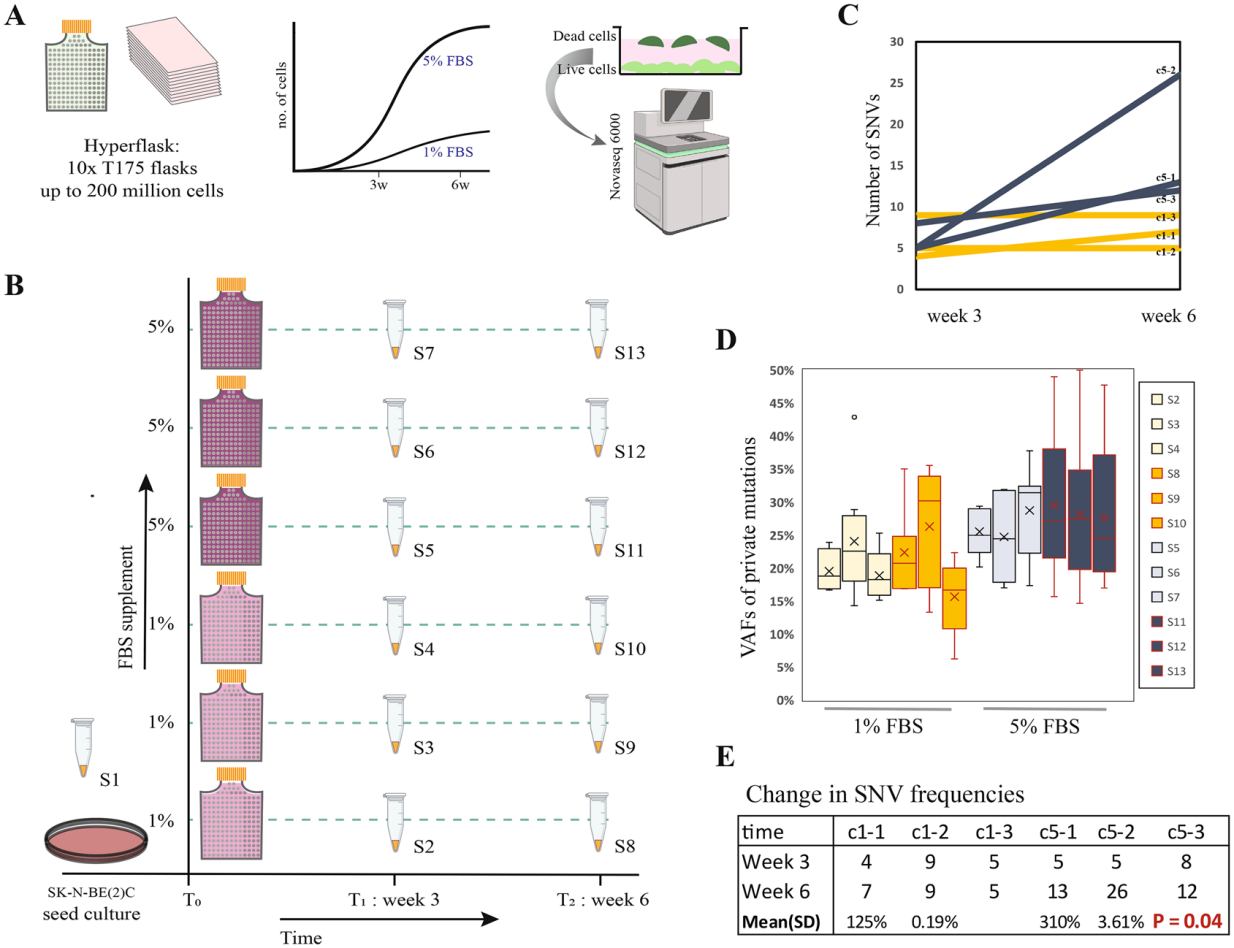
Differential mutation aggregation in long term cell culture with HyperFlask. (**A**) The Corning® HYPERflask® is a cell culture vessel designed for high yield performance. It features a 10-layer system with a total growth area of 1720 cm^2^. The surface of the flask is made of gas-permeable polystyrene, allowing it to support the growth of approximately 150 to 200 million cells. Neuroblastoma cell line SK-N-BE2c was expanded with 1% or 5% FBS in long term cultures for 6 weeks. Cell pellets were collected at the beginning of the experiment, after 3 weeks and at conclusion.Extracted DNA were subject to whole genome sequencing. (**B**) Seed culture was expanded to approximately 30 million cells and a cell pellet from this culture was saved (S1). Six HyperFlasks were seeded with 5 million cells each at the same time point. Three were supplemented with 1% FBS growth media and three with 5%. At the 3 week from each flask. (S2, S3, S4: 1% culture, S5, S6, S7: 5% culture). After 6 weeks the experiment was concluded, and cells were harvested (S8, S9, S10: 1% culture, S11, S12, S13: 5% culture). (**C**) Total number of SNVs detected at week3 and week6 for each culture is shown. (C1-1: S2&S8; C1-2: S3&S9; C1-3: S4&S10, C5-1: S5&S11; C5-2: S6&S12; C5-3: S7&S13) (**D**) Bar plots of VAFs for detected mutations in each timepoint are shown clustered according to growth media i.e., 1% or 5% FBS. ‘X’ signifies the mean. (**E**) Table depicting frequency of SNVs detected, aggregate and standard deviation per culture group.

To mimic the evolutionary dynamics of large populations (to accelerate evolutionary timeframe), we utilized a HYPERflask® (HF) cell culture system for adherent cells (Fig. 2A). This system allows each flask to hold up to 150–200 million cells, which is approximately 10 times more than a standard T175 flask. We seeded 5 million BE cells from a single culture (hereafter referred to as seed culture) in six HFs. We know from previous experiments that BE cells have markedly different growth dynamics when cultured in media supplemented with varying concentration of fetal bovine serum (FBS). We determined that for a growth medium supplemented with 1% FBS, BE cells demonstrate a logistic growth rate of approximately 1 and that for a 5% FBS media is well over 3 (Supplementary Fig. 31). Since these growth rates lie on two sides of the first bifurcation rate, we anticipated to recapitulate the differential growth under the two growth conditions by means of variability in mutation accumulation.

Cell pellets collected at the 3-week and at the 6-week (end of experiment) time points were subjected to whole genome sequencing; the seed culture was used as reference to call accumulated variants (Fig. 2B). Among the HF replicates, after 3 weeks both the 1% and the 5% cultures had acquired 6 novel single nucleotide variants (SNVs) on average. After 6 weeks this was 7 for the 1% culture whereas it increased to 20 SNVs on average for the 5% culture. The within group variance was quite low for the former at 0.19 but as high as 3.61 for the latter. This indicated not only a high rate of accumulation of mutations but also a large variation at rapid growth, consistent with the hypothesis of non-linear unpredictability; between group variation was statistically significant with a two/way ANOVA tested with two-tailed p value at 0.04 (Fig. 2C). Then we investigated the change in variant allele frequency (VAF) distribution between the two time points within and between groups, since a shift in VAF distribution may indicate change in fitness landscape (ex. Clonal sweep, fixation). We did not find any statistically significant difference, and VAFs of all groups clustered unimodally around 0.25 as expected (Fig. 2D-E; Supplementary table 10–12).

Since the seed culture was used as a baseline genome in these experiments, one concern was that subclonal mutations in the seed culture that would later increase in frequency and contribute the clonal landscape, would be filtered out, in turn resulting in a biased mutational landscape. We therefore released the baseline variant cut off to include variants with a VAF up to 0.1 in the seed culture (Supplementary Fig. 32). The larger variant pool was then tested with two-way ANOVA to detect varying mutation accumulation. This analysis was performed chromosome-wise and only those chromosomes were included for which at least 4 variants were detected across all samples. The 5% culture again had a statistically significant larger variation across cultures (two tailed p value 0.03), compared to the 1% culture. More telling was the between group sum of square estimate, which for the 1% culture was 0.007 compared to 0.11 for the 5% culture (Supplementary table 13).

## Discussion

Simulations of fast tumor growth (growth rates > 3.0) resulted in massively varied mutational landscapes, implying that evolutionary trajectories, even in controlled model systems, are unpredictable under conditions that invoke non-linearity. We simulated the population expansion process with a discrete growth following a logistic equation. This enabled us to implement an analytical approach to spatial simulation, but to some degree restricted genetic evolution as the population numbers reached at each generation were set to follow a logistic curve. A model of “genome chaos” has been used to describe non-continuous accumulation of genetic lesions during cancer evolution^27^. This model claims that tumor evolution is bi-phasic, starting with punctuated accumulation of mutations followed by a maintenance phase. The phase transition happens periodically based on changes in environmental factors also determining the rate at which the tumor grows/evolves. The co-dependence of the phases has been argued to result in macroevolutionary chaos originating from a predictable microevolutionary background^28^. Our experimental data imply that non-linear evolution is particularly likely to occur in fast-growing (pediatric) tumors. The mouse model data demonstrate that certain tumor types exhibit distinct growth characteristics, such as non-logistic or logistic growth patterns and slow or fast growth rates. These characteristics determine whether these tumors evolve clonally in an unpredictable or predictable manner. While the correlative evidence suggests a link between logistic growth rates exceeding the threshold of bifurcating states and complex genomic changes, further validation is necessary to establish a definitive causal relationship.

Our simulations model tumor expansion beginning from a small population (N₀ ≪ C), emulating early tumor growth below carrying capacity. This differs from systems at equilibrium (e.g., Fig. 1B) where population dynamics are governed by saturation effects. The initial conditions, whether in silico (N₀ ≈ 10^5^ cells) or in biological models (small xenograft inoculation/culture seeding) ensure growth occurs primarily in the exponential phase of the logistic curve, where stochastic mutations accumulate without density-dependent constraints.

In our cellular lineage experiments, we observed a distinct correlation between the growth rates and the accumulation of mutations. As the 5% FBS culture expanded at an accelerated pace compared to the 1% FBS culture, the predictability of mutational accumulation diminished. This heightened mutation accumulation, however, did not influence the clonal diversification of the culture, as evidenced by the unchanged VAF distribution. This allowed us to rule out alterations in the fitness landscape as the cause, suggesting that the observed effects on mutation predictability were emergent and were neutral from an evolutionary standpoint. Remarkably, this variation was discernible even at the chromosomal level, implying a potential genome-wide surge in stochasticity. Our observation underscores the intricate dynamics of cellular evolution and the profound impact of growth rates on mutation accumulation within a population. It also highlights the importance of considering factors such as growth rates and its impacts on mutational accumulation when interpreting experimental results in cellular biology and genomics.

The focus of this study was to investigate the possible relationship between growth rates of tumor model system and corresponding mutational changes leading to selective pressure and evolutionary adaptation. However, there are several other mechanisms that can modulate the expression and activity of genes and proteins notwithstanding point mutation such as structural variations and large-scale karyotype changes. Such effects may also arise without alteration in the DNA sequence such as epigenetic changes and in the form of transcriptional phenotypic cell states. Furthermore, the tumor microenvironment can provide signals that promote tumor growth, angiogenesis, invasion, metastasis, and modulate the immune environment. Consequently, it would be reductive to infer the behavior of a model system solely based on genetic point mutations. Muller’s ratchet is a phenomenon in evolutionary genetics where the accumulation of deleterious mutations in an asexual population leads to a gradual decline in fitness. While this is typically associated with deleterious mutations, our study explores how neutral mutations can also contribute to fitness decline through a process called accumulation. In the absence of direct deleterious effects, neutral mutations can accumulate over time, leading to a decrease in overall genetic fitness. Additionally, we acknowledge that the normalized time-scale employed in our analysis across model systems may not be evolutionarily identical, as doubling times vary among them. We cannot speculate whether this variation itself can drive differential evolution, similar to Peto’s paradox. Furthermore, we generalized the model system by fitting the logistic curve, whereas some literature suggests that the aggressiveness of certain cancers may render them suitable fits to accelerated time models. However, pediatric PDX tumors and in vitro systems would not be appropriate fits for such models.^29^. Notwithstanding the choice of the model, we also ignore environmental constraints of the growth that will certainly exert selective pressure such as necrosis, angiogenesis, induction of hypoxia, changes in micro environment and cellular phenotypes.

Several studies have modeled in-vivo tumor growth using nonlinear differential equations, which often exhibit chaotic behavior^30^. This infers that small changes in initial conditions can lead to vastly different outcomes, making the system highly sensitive and unpredictable. For example, these efforts have revealed some interesting things, like how tumors can double in size and become unpredictable, or how they can have different states at the same time highlighting complexity and unpredictability.^31^. Additionally nutrient driven models also show that slight changes in initial conditions may lead to chaotic expansion with embedded local attractors^32^. For instance, the fractal nature of tumor growth has been documented, indicating self-similarity and scale-invariance, which are hallmarks of complex systems^31^. Additionally, the presence of chaotic attractors in tumor growth models further supports the idea that tumors can exhibit unpredictable and complex behavior^32^. In mechanistic terms the often exhibition high levels of genetic instability is a derivative of accelerated local growth. This instability allows for rapid and unpredictable changes in the tumor genome, leading to a diverse population of tumor cells with varying characteristics^33^. Genetic instability relates to local-scale growth, while selective pressures maintain global stability, creating a dynamic and unpredictable system. The tumor microenvironment plays a crucial role, with interactions between tumor cells and their environment leading to nonlinear dynamics. Nutrient availability, immune response, and cellular signaling pathways contribute to complexity and unpredictability of tumor growth^34^.

Our study utilized the proportion of MRCA cells as a simplified measure of clonal heterogeneity, based on the assumption that genetic distance from ancestral populations reflects evolutionary divergence. However, this metric does not account for the spatial organization of clusters or the number of distinct subclonal populations. Tumors are spatially structured ecosystems where subclones may occupy distinct niches, influencing their access to resources, interactions with the microenvironment, and competitive dynamics. For instance, even a low proportion of MRCA cells could mask significant heterogeneity if spatially segregated clusters exhibit divergent evolutionary trajectories. Conversely, a high MRCA proportion might coexist with localized clusters of aggressive subclones poised for expansion. The number of clusters and their spatial distribution introduce additional layers of complexity. A tumor with many small, genetically distinct clusters may exhibit higher functional heterogeneity than one dominated by a few large clusters, even if their MRCA proportions are similar. Spatial separation could also buffer subclones from competitive exclusion, enabling coexistence and increasing the potential for unpredictable clonal sweeps. Our simulations, which abstracted spatial constraints into a logistic growth framework, did not explicitly model these effects. Thus, the MRCA proportion may underestimate heterogeneity in systems where spatial clustering promotes subclone persistence or accelerates adaptation.

Going forward, evolutionary dynamics for tumors growing in a non-logistic fashion need also to be compared alongside logistically growing tumors/cell populations to evaluate if the unpredictability in genetic mutation accumulation is specific to all non-linear growth. According to the punctuated evolutionary model for cancer^27^, the maintenance phase acts as a self-regulatory mechanism to reorganize cells after a period of arbitrary mutation accumulation. It’s possible that the observed unpredictability stems from the dysregulation in these two phases of evolution, which disrupts the natural equilibrium of evolutionary homeostasis. There seems to be an intrinsic limit on how much of the genetic changes may be predicted using today’s approach of precision oncology. However, our findings indicate that observing the tumor growth rate could be a potential workaround to determine the degree of predictability by which a tumor is expected to evolve, in turn providing insights into how often the clonal landscape of a tumor needs to be resampled to evaluate options for targeted therapy based on the molecular profile.

## Methods

### Assumptions underlying in silico modeling

Here, we simulated a synthetic model for tumorigenesis, incorporating some of the basic assumptions of the somatic mutation theory. It remains to be seen whether DNA damage-driven neutral or Darwinian evolution becomes apparent with this system, as the model does not assume a position on either dogma of continuous tumor evolution. The model focuses on simulating evolutionary pockets in a tumor soma that vary across its geography, similar to how it is explained by Waclaw and colleagues^12^. A scheme of the iterative model is sketched in Supplementary Fig. 33.

We begin the simulation with a predetermined number of cells, C, indicating a fixed carrying capacity (maximum number of cells in homeostasis, assumed to be 10 million) in a three-dimensional automaton where all but a few (randomly chosen, < 10) neighboring cells are considered to be non-neoplastic at the beginning (generation 0). Over the total span of each simulation, we modulated the following five properties and treated them as simulation parameters:

- Cell division rate/birth rate (**b**)
- Cell death rate (**d**)
- Mutation rate (**m**)
- Driver mutation probability (**p**)
- Selective advantage/fitness (**s**)

These parameters in tandem control the size of the tumor, how fast it grows, and how genetically diverse and evolved it is at the end of the simulation. As the automaton simulates cellular interaction and not the expansion magnitude, we iterated the evolutionary simulation over punctuated time points. The generalized continuous form is given as follows:

1 $$dN/dt=r{N}_{t}(1-{N}_{t}/C)$$

The population size is iterated over a discrete logistic growth solved analytically as a function of discrete time. Given fixed doubling time of the cells, resource abundance dependent negative feedback of the population frequency and C >  > N_0_, Eq. (1) can be simplified as following^35^ where C is carrying capacity of the system, ${N}_{t}$ is the number of tumor cells at any given time $t$, and $r$ is the growth rate.

2 $${N}_{t}= \frac{\text{C}{N}_{0}}{{N}_{0}+(\text{C}-{N}_{0}){e}^{-rt}}$$

We estimated ${N}_{t}$ iteratively for time $t=\text{0,1},2,\dots$ proxied by the set of whole numbers and simulated the discretized evolutionary dynamic for a tumor expanding from ${N}_{0}$ cells to ${N}_{t}$ cells. The time scales were normalized to a scale of 0 to 1 with equipartition steps of growth for each model across all replicates (1,000). We implemented these dynamics in the simulation by assuming certain base hypotheses.

The ratio of tumor size at each step (${N}_{t}$) to carrying capacity (C) significantly impacts simulated tumor growth. When N is small, the simulation models rapid growth due to abundant resources. As N approaches C, resource limitation slows growth. If N equals C, the tumor may outgrow resources, potentially experiencing a “boom and bust” cycle or accessing new resources. These growth phases affect mutation accumulation, clonal expansion, and evolutionary trajectories. In the accelerating phase, mutation accumulation may be faster due to less selective pressure. In the decelerating phase, dominant clones best suited for limited resources may emerge.

1. Mutational burden is predicted by the type of mutation and the mutation rate.

At each division any given daughter cell acquires a mutational burden quantified by D_n_; n ∈ ℕ. The number of neoplastic cells, n, in the automata dictates when the simulation ends, that is, when n = N. Hence, we leverage it as a proportion of time. Since D_n_ changes depending on the mutation rate, and on the effect the accumulated mutations have on the cell, it follows that D_n_ is a function of both **m** and **p** as described above.

D_n_ = f (m, p); m ∈ R^+^ and p ∈ (0,1)

2. Cell birth and death rates depend on mutational burden.

Every k^th^ cell in the population undergoes cell division after time T (k_birth, n_) and apoptosis after time T (k_death, n_); n ∈ ℕ, k ∈ ${\mathbb{Z}}^{+}$. These are proxies for *birth rate* and *death rate* and, therefore, must functionally depend on the acquired mutational burden.

T (k_birth, n_) = f_birth_ (D_n_)

T (k_death, n_) = f_death_ (D_n_)

Acquiring certain mutations creates a positive feedback loop.

With this, we assume that the probability of any cell acquiring mutational burden is predicated by pre-existing lesions in its DNA. At any i^th^ cell division, the probability of a cell acquiring additional mutational burden is dependent on its preexisting burden after the (i − 1)^th^ cell division. Because the cumulative mutational load defines the selective advantage that a cell harbors at any given point,

s_i_ = f (s_i-1_), where s_i_ and s_i-1_ are the selective advantages of a cell at each stage and take the functional form of a Markov process, respectively. The selection coefficient is thus shaped indirectly by mutational burden.

### Modeling acquisition of mutations

In this study, we considered only somatic point mutations extracted from publicly available datasets. This is in no way to indicate a lack of availability of data on structural chromosomal changes as well as longer variations, but only due to the unsubstantiated mechanics of conferring selective advantage or disadvantage. We also did not consider inherited mutations, as all cells should harbor the same set of variations, which in turn should have scalable effects across the entire cell population. How these factors affect the evolutionary process is of course of interest, but we assume that the point mutations do not affect the relative fitness of the cells. Hence, we adapted a system of evolutionary dynamics that is only affected by point mutations, and to this end, we used their pathogenicity scores.

We extracted the COSMIC v90 dataset (https://cancer.sanger.ac.uk/cosmic/download) for all reported point mutations along with the estimated pathogenicity scores (FATHMM-MKLv2.3)^36^. Every mutation was given a score with a lower limit of 0 and an upper limit of 1 with respect to the corresponding cancer site and histology when applicable. COSMIC identifies strong drivers with score stratification which we use to stratify all possible mutations into two groups, *neutral and pathogenic*. A FATHMM score between 0.5 and 0.7 indicates a ‘driver’ and that above 0.7 indicates a ‘strong driver’. We assumed a mutation to be pathogenic if it had a score > 0.5 and the rest were declared neutral. We extracted the pathogenicity scores of only the pathogenic mutations to compute the fitness advantage. By definition, fitness is indifferent to neutral mutations, yet these were detected in the genetic background. The overaccumulation of neutral mutations caused the cell division machinery to halt above a critical threshold (discussed below), although it did not directly impact cellular fitness.

The fitness landscape was shaped based on two fundamental principles. Firstly, all site-specific driver mutations reported to date have been identified in the literature, as well as those associated with individual hallmarks of cancer. These mutations are extracted for tumor simulation at those specific sites. We retained drivers reported in neuroblastoma as the number of driver mutations and their strength of adding to the fitness advantage would determine how the clonal geography takes shape. Once such a mutation is acquired by a cell, it confers a selective advantage to cell survival according to its pathogenicity score which got updated progressively. Second, neutral mutations confer mutational burden only via accumulation such that their cumulative accumulation over generations triggers apoptosis akin to Muller’s ratchet^37^.

### How neutral mutations affect growth

We take that the number of neutral mutations will increase over generations. Hence, the carrying capacity of the population with a given number of mutations is affected, which is given by the equation

3 $$n_{k} = {\text{N}}e^{{ - \theta }} \theta ^{k} /k!$$

where θ =* S*/*m*. Here, n_k_ is the carrying capacity of the cell population harboring neutral mutations, where N is the total population size, m is the mutation rate, and s is the corresponding selection coefficient/fitness. Hence, it can be concluded that the population size-scaled carrying capacity follows a Poisson distribution:

4 $$\frac{{n}_{k}}{N}=Poisson\left(\theta \right)$$

over the random variable K assuming values k ∈ N^+^ and the carrying capacity of the fittest class of cells, that is, at the beginning of simulation is given by

5 $${n}_{0}=\text{N}{e}^{-\uptheta }$$

No technical replication was completed because the summaries were extracted over multiple runs of simulations. The simulations were performed with SITH^12^; prediction, fitting and evaluation of the models were performed in R version 4.1.

### Growth modulated cell culture in HYPERflasks

We cultured the SK-N-BE(2)C neuroblastoma cell line (passage 14 onwards, obtained from DSMZ) supplemented with 1% or 5% FBS (Sigma-Aldrich) and 1% penicillin–streptomycin (Sigma-Aldrich). The culture was confirmed to be mycoplasma free (Eurofins). The cells were expanded in High Yield PERformance Flasks (HYPERflask®) cell culture vessel (Corning). Growth media was changed once every week. Cells were resuspended with TrypLE™ Express Enzyme (Thermo Fisher) and were harvested on stipulated experiment end points. Cell viability was measured at harvest with Trypan blue exclusion assay (Thermo Fisher) and counted with Countess™ platform (Thermo Fisher).

The in-vitro models had a maximum capacity of around 150 million cells due to the limitations of the HyperFlask systems used. Here the initial volumes were estimated to 5 million cells. For the mice models, the volume of the tumor growing peritoneally in the animals was capped by the ethical permit, allowing a range of 1500 to 5000 cubic millimeters of total tissue volume across different studies.

### DNA extraction and Bulk sequencing

DNA from frozen SKNBE2C cell pellets were extracted using the AllPrep DNA/RNA Mini Kit 127 (Qiagen) with standard methods. DNA concentration was measured using Qubit DS HS DNA 132 (Invitrogen).

Whole genome sequencing of thirteen samples were performed at the Center for Translational Genomics at Lund. Sequencing libraries of genomic DNA were prepared using the TruSeq DNA PCR-free Library Prep kit (Illumina) following the manufacturer’s instructions and quality controlled with TapeStation 4200 (Agilent). The libraries were pooled and subjected to (2 × 150 cycles) paired-end sequencing with the NovaSeq 6000 platform (Illumina).

bcl2fastq 2.20 (Illumina) was used to convert files to fastq-formatted paired end files, the reads were quality controlled with *fastqc*, adapters were adjusted with *cutadapt* and the reads were mapped to the human reference genome (GRCh38 v3) using *strobealign*. Duplicate reads were marked with *sambamba*^38^(–overflow-list-size 6,000,000 –hash-table-size 6,000,000 –sort-buffer-size 102,400) and the reads were sorted, indexed with the same. The mapped reads were further normalized with *GATK BQSR* (known site flags: 1000G_omni2 for hg38, HapMap_3 for hg38, hg38 known indels and Mills_and_1000G_gold_standard indels)^39^.

Somatic variant calling was performed using four separate variant callers with the same reference and target, Mutect2, Varscan2, Octopus and Strelka2^40–43^. Structural variants were called with Manta and copy numbers were analyzed against a normal ancestrally distant human sample obtained from GiAB HG006 and the seeding cell pallet sequenced. Variants were first annotated with *BCFTools* and filtered with *GATK* (orientation bias, mapping quality and segmentation bias) and were subsequently excluded from downstream analysis if detected with 10 or less reads, with a variant allele frequency (VAF) below 0.1 in all samples or if found in more than 1% of the reads in the seeding sample. The variants were then functionally annotated with *Annovar* merging annotations from ClinVar 20,221,232, COSMIC v97, dbSNP v42, ExAC v3, ICGC v28, ljb v26, GNOMAD, ENSEMBL and refGene.

We confirmed the identity of the SK-N-BE(2)C cells by comparing the copy number profiles against previously published profiles. When grown in 10-cm petri dishes these cells show an ecological threshold of how much they can expand which provides estimates of carrying capacity under each growth condition, this was used to estimate the growth rates.

## Supplementary Information

Supplementary Information.

## Data Availability

All PDX data are published or available from the respective corresponding authors and are detailed in Supplementary Table 1. WGS data are available from EGA under the accession (EGAS00001007962).
