# Supplementary material for: Evolutionary unpredictability in cancer model systems
Source: Sci Rep. 2025 Jun 27;15:20334. doi: 10.1038/s41598-025-07407-6 (PMC12205069; doi:10.1038/s41598-025-07407-6)

Supplementary Table 1. Summary of accumulated experiments

**Author identifier**	**Cancer type**	**sample type**	**No. of replicates**	**Total no. of mice**	**Source**
Hansson et. al.	Neuroblastoma	PDX	2	11	https://doi.org/10.1126/scitranslmed.aba4434
Radke et. al.	Neuroblastoma	PDX	1	7	https://doi.org/10.1016/j.tranon.2021.101149
Mohlin et. al.	Neuroblastoma	cell lines	5	32	https://doi.org/10.1158/0008-5472.CAN-15-0708; (some unpublished)
Manas et. al.	Neuroblastoma	PDX	6	30	https://doi.org/10.1101/2022.04.01.486670
Murphy et. al.	Wilms tumor	PDX	6	48	https://doi.org/10.1038/s41467-019-13646-9
n.a.	Breast cancer	cell lines	2	18	Unpublished data
n.a.	Lung cancer	cell lines	5	34	Unpublished data

Supplementary Table 2. Neuroblastoma PDXs (Hansson et. al.)

Replicate 1.

Control	Growth rate	%log	%chaos
**A1**	2.9	75%	67%
**A2**	6.9		
**A3**	7.1		
**A4**	n.a		

Replicate 2.

Control	Growth rate	%log	%chaos
**A1**	4.1	43%	100%
**A2**	n.a		
**A3**	n.a		
**A4**	4.4		
**A5**	n.a		
**B1**	4.9		
**B2**	n.a		

%log: Percentage of mice that followed a logistic growth
%chaos: Percentage of mice following logistic growth that observed growth rate over 3.0

Supplementary Table 3. Neuroblastoma PDXs (Radke et. al.)

Control	Growth rate	%log	%chaos
**A1**	n.a	43%	67%
**A2**	2.5		
**A3**	n.a		
**A4**	9.8		
**A5**	n.a		
**A6**	n.a		
**A7**	8.8		

%log: Percentage of mice that followed a logistic growth
%chaos: Percentage of mice following logistic growth that observed growth rate over 3.0

Supplementary Table 4. Neuroblastoma SK-N-BE2(C) cell line (Mohlin et. al.)

Replicate 1.

Control	Growth rate	%log	%chaos
**A31**	4.8	50%	80%
**A41**	6.2		
**B11**	n.a		
**B21**	n.a		
**B31**	5.8		
**A32**	1.7		
**A42**	11		
**B12**	n.a		
**B22**	n.a		
**B32**	n.a		

Replicate 2.

Control	Growth rate	%log	%chaos
**A1**	1.4	60%	67%
**A2**	3.7		
**A3**	4		
**A4**	n.a		
**A5**	n.a		

Replicate 3.

Control	Growth rate	%log	%chaos
**m1**	n.a	60%	100%
**m2**	6.5		
**m3**	20		
**m4**	n.a		
**m5**	15		

Replicate 4.

Control	Growth rate	%log	%chaos
**m1**	5	44%	75%
**m2**	n.a		
**m3**	n.a		
**m4**	n.a		
**m5**	n.a		
**m6**	n.a		
**m7**	1.5		
**m8**	5.6		
**m9**	3.7		

Replicate 5.

Control	Growth rate	%log	%chaos
**m1**	3.9	67%	100%
**m2**	n.a		
**m4**	4.1		

%log: Percentage of mice that followed a logistic growth
%chaos: Percentage of mice following logistic growth that observed growth rate over 3.0

Supplementary Table 5. Neuroblastoma PDXs (Manas et. al.)

Model: PDX1 (nude mice)

Control	Growth rate	%log	%chaos
**c1**	4.5	75%	100%
**c2**	3.1		
**c3**	n.a		
**c4**	11		

Model: PDX1 (NSG mice)

Control	Growth rate	%log	%chaos
**c1**	25	60%	100%
**c2**	29		
**c3**	n.a		
**c4**	n.a		
**c5**	6		

Model: PDX2 (NSG mice)

Control	Growth rate	%log	%chaos
**c1**	4.4	71%	100%
**c2**	13		
**c3**	11		
**c4**	n.a		
**c5**	16		
**c6**	12		
**c7**	n.a		

Model: PDX3 (nude mice), replicate 1

Control	Growth rate	%log	%chaos
**C1**	1.8	50%	50%
**C2**	6.4		
**C3**	n.a		
**C4**	n.a		

Model: PDX3 (nude mice), replicate 2

Control	Growth rate	%log	%chaos
**c1**	n.a	40%	100%
**c2**	9.4		
**c3**	n.a		
**c4**	9.8		
**c5**	n.a		

Model: PDX3 (nude mice), replicate 3

Control	Growth rate	%log	%chaos
**c1**	17	80%	100%
**c2**	18		
**c3**	n.a		
**c4**	16		
**c5**	16		

%log: Percentage of mice that followed a logistic growth
%chaos: Percentage of mice following logistic growth that observed growth rate over 3.0

Supplementary Table 6. Wilms tumor PDXs (Murphy et. al.)

Model: KT47

Control	Growth rate	%log	%chaos
**m1**	n.a	75%	100%
**m2**	30		
**m3**	18		
**m4**	34		
**m5**	32		
**m6**	40		
**m7**	n.a		
**m8**	27		

Model: KT53

Control	Growth rate	%log	%chaos
**m1**	31	100%	100%
**m2**	35		
**m3**	37		
**m4**	34		
**m5**	32		
**m6**	32		
**m7**	27		
**m8**	32		

Model: KT51

Control	Growth rate	%log	%chaos
**m1**	21	88%	100%
**m2**	6.8		
**m3**	15		
**m4**	15		
**m5**	40		
**m6**	n.a		
**m7**	39		
**m8**	15		

Model: KT45

Control	Growth rate	%log	%chaos
**m1**	n.a	63%	100%
**m2**	n.a		
**m3**	5.8		
**m4**	20		
**m5**	12		
**m6**	4.3		
**m7**	7.3		
**m8**	n.a		

%log: Percentage of mice that followed a logistic growth
%chaos: Percentage of mice following logistic growth that observed growth rate over 3.0

Model: KT75

Control	Growth rate	%log	%chaos
**m1**	n.a	38%	100%
**m2**	n.a		
**m3**	8.2		
**m4**	4.8		
**m5**	n.a		
**m6**	n.a		
**m7**	n.a		
**m8**	7.6		

Model: KT43

Control	Growth rate	%log	%chaos
**m1**	n.a	13%	100%
**m2**	n.a		
**m3**	4.4		
**m4**	n.a		
**m5**	n.a		
**m6**	n.a		
**m7**	n.a		
**m8**	n.a		

%log: Percentage of mice that followed a logistic growth
%chaos: Percentage of mice following logistic growth that observed growth rate over 3.0

Supplementary Table 7. Breast cancer cell line models

Cell line: MCF7

Control	Growth rate	%log	%chaos
**m1**	0.9	89%	0%
**m2**	0.23		
**m3**	0.98		
**m4**	0.63		
**m6**	n.a		
**m7**	0.97		
**m8**	0.99		
**m9**	0.98		
**m10**	0.87		

Cell line: MDA-MB-231

Control	Growth rate	%log	%chaos
**m1**	1.5	100%	11%
**m2**	2.9		
**m3**	3.6		
**m5**	0.52		
**m6**	2.80		
**m7**	1.20		
**m8**	1.30		
**m9**	2.50		
**m10**	1.30		

%log: Percentage of mice that followed a logistic growth
%chaos: Percentage of mice following logistic growth that observed growth rate over 3.0

Supplementary Table 8. Lung cancer cell line models

Cell line: A549 (replicate 1)

Control	Growth rate	%log	%chaos
**m1**	0.7	88%	0%
**m2**	0.35		
**m3**	0.9		
**m4**	0.032		
**m5**	0.66		
**m6**	0.99		
**m7**	0.74		
**m8**	n.a		

Cell line: A549 (replicate 2)

Control	Growth rate	%log	%chaos
**m1**	0.37	100%	0%
**m2**	0.6		
**m3**	0.93		
**m4**	0.62		
**m5**	0.33		
**m6**	0.25		
**m7**	0.64		
**m8**	0.49		

Cell line: H520 (replicate 1)

Control	Growth rate	%log	%chaos
**m1**	n.a	60%	0%
**m2**	1.1		
**m3**	2		
**m4**	n.a		
**m5**	1.6		

Cell line: H520 (replicate 2)

Control	Growth rate	%log	%chaos
**m1**	n.a	40%	0%
**m2**	1.60		
**m3**	1.70		
**m4**	n.a		
**m5**	n.a		

Cell line: H441

Control	Growth rate	%log	%chaos
**m1**	0.65	75%	0%
**m2**	0.29		
**m3**	n.a		
**m4**	1.7		
**m5**	2.1		
**m6**	1.6		
**m7**	0.33		
**m8**	n.a		

%log: Percentage of mice that followed a logistic growth
%chaos: Percentage of mice following logistic growth that observed growth rate over 3.0

Supplementary Table 9. Summary of growth rates across experiments. The growth rates are accumulated for all mice that adhered to logistic pattern. All growth rates more than 3 are color coded in orange to reflect possible mechanisms of chaos in those mice and all less than 3 are shown in green as these are unlikely to exhibit chaotic fluctuations. This heatmap provides an overview for possible inclination of cancer types under consideration to observe chaotic growth.

Source	Cancer type	Growth rates								
Hansson et.al.	Neuroblastoma	4.1	4.4	4.9						
	Neuroblastoma	2.9	6.9	7.1						
Radke et.al.	Neuroblastoma	2.5	9.8	8.8						
Mohlin et.al.	Neuroblastoma	4.8	6.2	5.8	1.7	11				
	Neuroblastoma	1.4	3.7	4						
	Neuroblastoma	5	1.5	5.6	3.7					
	Neuroblastoma	6.5	20	15						
	Neuroblastoma	3.9	4.1							
Manas et. al.	Neuroblastoma	4.5	3.1	11						
	Neuroblastoma	25	29	6						
	Neuroblastoma	4.4	13	11	16	12				
	Neuroblastoma	1.8	6.4							
	Neuroblastoma	9.4	9.8							
	Neuroblastoma	17	18	16	16					
Murphy et.al.	Wilms tumor	30	18	34	32	40	27			
	Wilms tumor	31	35	37	34	32	32	27	32	
	Wilms tumor	21	6.8	15	15	40	39	15		
	Wilms tumor	5.8	20	12	4.3	7.3				
	Wilms tumor	8.2	4.8	7.6						
	Wilms tumor	4.4								
n.a.	Breast cancer	0.9	0.23	0.98	0.63	0.97	0.99	0.98	0.87	
	Breast cancer	1.5	2.9	3.6	0.52	2.80	1.20	1.30	2.50	1.30
	Lung cancer	0.7	0.35	0.9	0.032	0.66	0.99	0.74		
	Lung cancer	0.37	0.6	0.93	0.62	0.33	0.25	0.64	0.49	
	Lung cancer	1.1	2	1.6						
	Lung cancer	1.60	1.70							
	Lung cancer	0.65	0.29	1.7	2.1	1.6	0.33			

scale
<1
3
>4

Supplementary Table 10. Change in number of SNVs detected.

Time	c1-1	c1-2	c1-3	c5-1	c5-2	c5-3
Week 3	4	9	5	5	5	8
Week 6	7	9	5	13	26	12
%change	1.75	1	1	2.6	5.2	1.5
Mean(SD)	1.25	0.19		3.1	3.61	P = 0.04

Supplementary Table 11. Change in average VAF.

Time	c1-1	c1-2	c1-3	p-value
Week 3	0.20	0.24	0.19	
Week 6	0.22	0.26	0.16	P>0.5
	c5-1	c5-2	c5-3	
Week 3	0.26	0.25	0.29	
Week 6	0.30	0.28	0.28	P>0.5
Ovaerall	0.26			

Supplementary Table 12. Correlation in % of private mutations acquired.

time	c1-1	c1-2	c1-3	c5-1	c5-2	c5-3
private %	0.50	0.44	0.20	0.40	0.40	0.13
private %	0.57	0.22	0.80	0.15	0.58	0.25
%change	1.14	0.50	4.00	0.38	1.44	2.00
Spearman	0.64	-0.10				
T score	0.84	-0.21				
p-value	0.56	0.84				

Supplementary Table 13. Two-way ANOVA testing variations in chromosome-wide SNV aggregation

Anova: Single Factor — 1% Conc.

SUMMARY

Groups	*Count*	*Sum*	*Average*	*Variance*
Row 1	6	1.671057	0.27851	0.049742
Row 2	6	1.967787	0.327965	0.076466
Row 3	6	1.82162	0.303603	0.046043

ANOVA

Source of Variation	*SS*	*df*	*MS*	*F*	*P-value*	*F crit*
Between Groups	0.007338	2	0.004	0.06	0.94	3.68
Within Groups	0.861254	15	0.057			
Total	0.868592	17				

Anova: Single Factor — 5% Conc.

SUMMARY

Groups	*Count*	*Sum*	*Average*	*Variance*
Row 1	6	2.447343	0.40789	0.119142
Row 2	6	2.8062	0.4677	0.092166
Row 3	6	3.571029	0.595172	0.067312

ANOVA

Source of Variation	*SS*	*df*	*MS*	*F*	*P-value*	*F crit*
Between Groups	0.109801	2	0.05	0.59	0.57	3.68
Within Groups	1.393099	15	0.09			
Total	1.5029	17				

Anova: Two-Factor With Replication (1% vs 5%)

SUMMARY	chr1	chr10	chr11	chr13	chr21	chr6	Total
0.01							
Count	3	3	3	3	3	3	18
Sum	0.190357	1.027633	0.175295	1.433374	0.767139	1.866667	5.460464
Average	0.063452	0.342544	0.058432	0.477791	0.255713	0.622222	0.303359
Variance	0.002518	0.010062	0.001579	0.031998	0.006481	0.001481	0.051094
0.05							
Count	3	3	3	3	3	3	18
Sum	1.559741	0.884105	1.953793	1.086308	1.534997	1.805629	8.824573
Average	0.519914	0.294702	0.651264	0.362103	0.511666	0.601876	0.490254
Variance	0.106977	0.081437	0.157852	0.123009	0.05221	0.088387	0.088406
Total							
Count	6	6	6	6	6	6	
Sum	1.750098	1.911738	2.129088	2.519683	2.302135	3.672296	
Average	0.291683	0.318623	0.354848	0.419947	0.383689	0.612049	
Variance	0.106305	0.037286	0.169208	0.066018	0.04313	0.036071	

ANOVA

Source of Variation	*SS*	*df*	*MS*	*F*	*P-value*	*F crit*
Sample	0.314367	1	0.314367	5.68141	**0.03**	4.259677
Columns	0.395767	5	0.079153	1.430501	0.25	2.620654
Interaction	0.647741	5	0.129548	2.341263	0.07	2.620654
Within	1.327983	24	0.055333			
Total	2.685859	35				

Supplementary figure legends

Supplementary figure 1-4.

Prediction of growth trend (red if logistic, green if not) are shown for each growth curve, experiment wise. For neuroblastoma, **a-e** are from Mohlin et. al., **f-g** are from Hansson et. al., **h** is from Radke et. al. and, **i-n** are from Manas et. al. The Wilms tumor, breast and lung cancer samples are taken from sources as discussed in supplementary table 1. All samples are ordered as they appear in supplementary tables 2-8.

Supplementary figures 5-30.

Good ness of logistic fit is shown with fitted curve and overlayed tumor volume measures. Each plot refers to a single control mouse belonging to a set of experiments whose growth adhered to a logistic growth. For example, mice from replicate 1 of Hansson et. al. are shown in supplementary figure 5. Supplementary table 2 for the replicate 1 shows three mice (A1, A2, A3) followed a logistic growth. In supplementary figure 5, goodness of fit are shown for these three mice. All plots accompany the mouse identifier that correspond to supplementary tables 2-8 for respective experiment.

Supplementary figure 31.

Neuroblastoma cell line SKNBE2C is cultured in triplicates supplemented with 1% or 5% FBS growth media. Each culture was seeded with 40,000 cells. The order of growth measures Xtimes of the number of live cells at any point normalized against seeding number. The carrying capacity of a culture was estimated at the peak number of live cells observed. Doubling times were empirically estimated.

Supplementary figure 32.

Chromosome specific VAF distributions of the temporally varying samples collected in the SKNBE2C cell culture experiment. Only chromosomes with at least 4 mutations detected with the relaxed variant calling condition across all samples are shown.

Supplementary figure 33.

Here we provide a schematics of the simulation dynamics. The discreet logistic function on top is plotted over normalized time of one whole unit. The minor Y-axes breaks show the equidistant time points when the total population estimates were retrieved (N_j). These were put to SITH in estimating tumor population with re-estimated parameters at each step based on the mutation burden contrived from deliteriousness scores.

Supplementary figure 1. **Neuroblastoma**

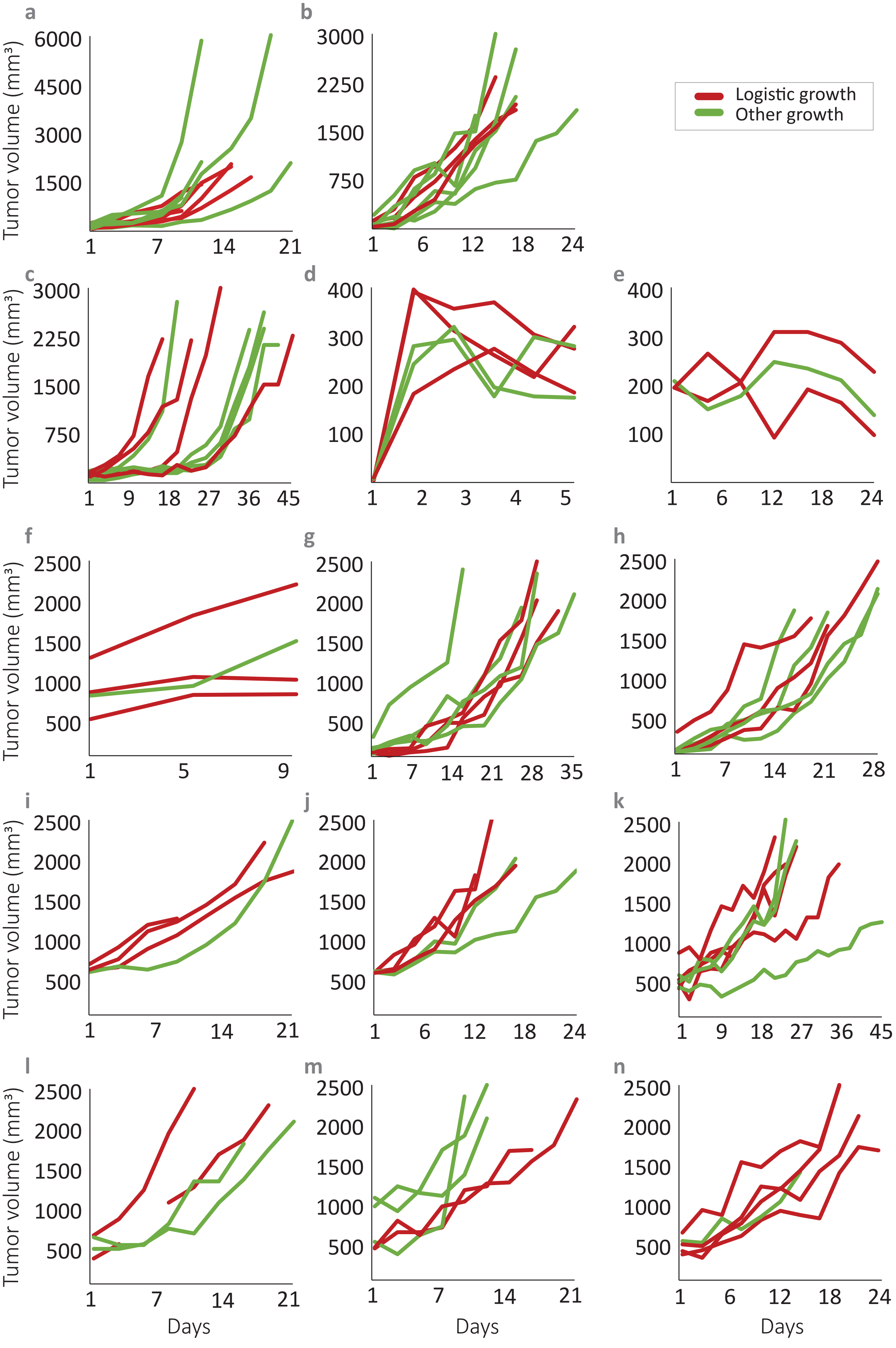

Supplementary figure 2. **Wilms tumor**

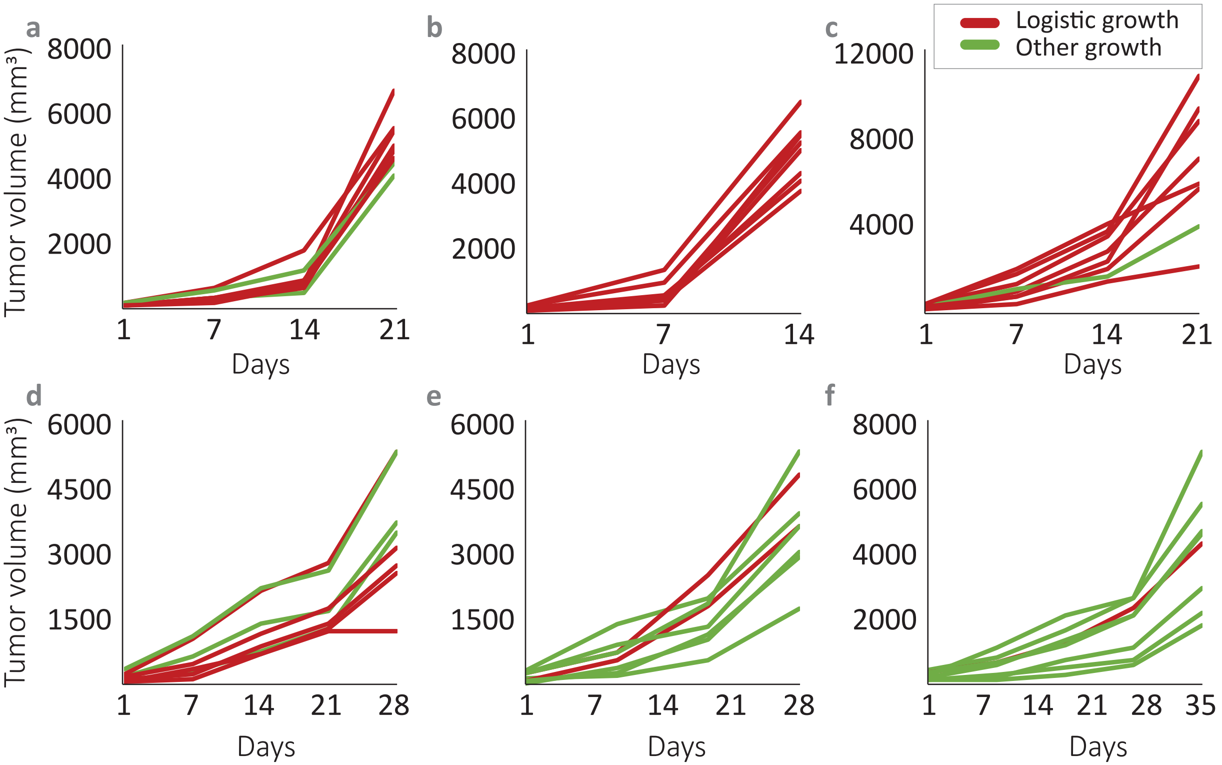

Supplementary figure 3. **Breast cancer**

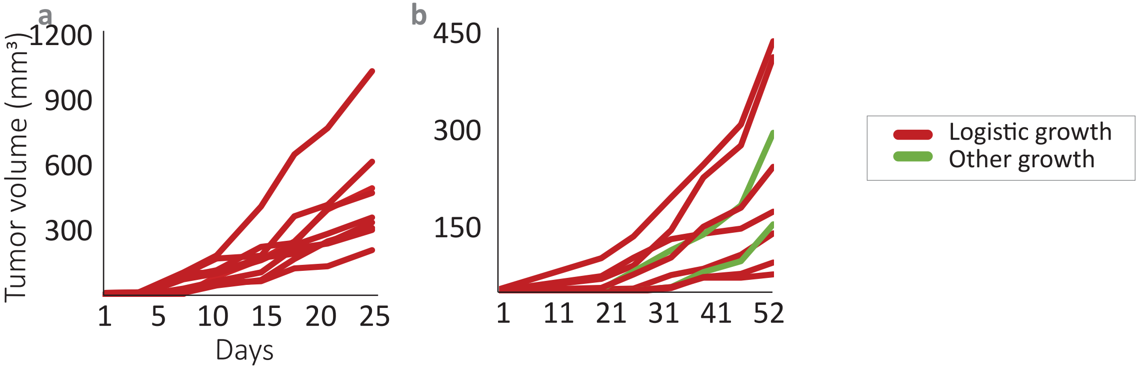

Supplementary figure 4. **Lung cancer**

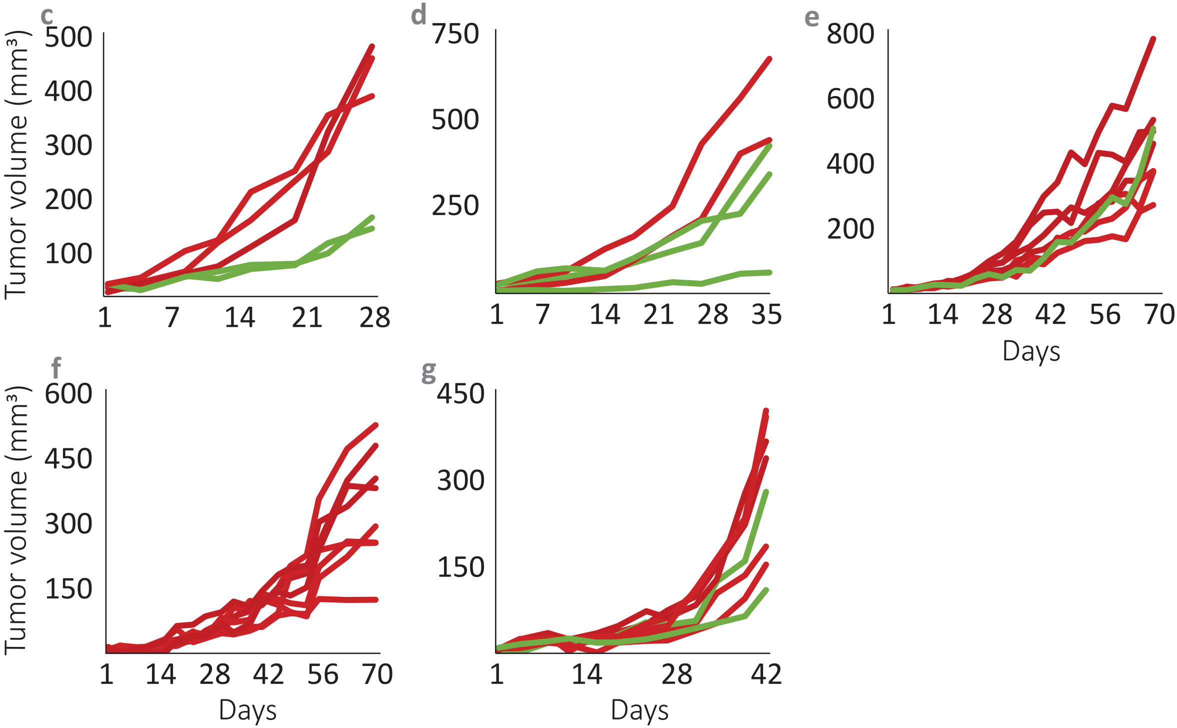

Supplementary figure 5. Neuroblastoma PDX (Hansson et. al., replicate 1)

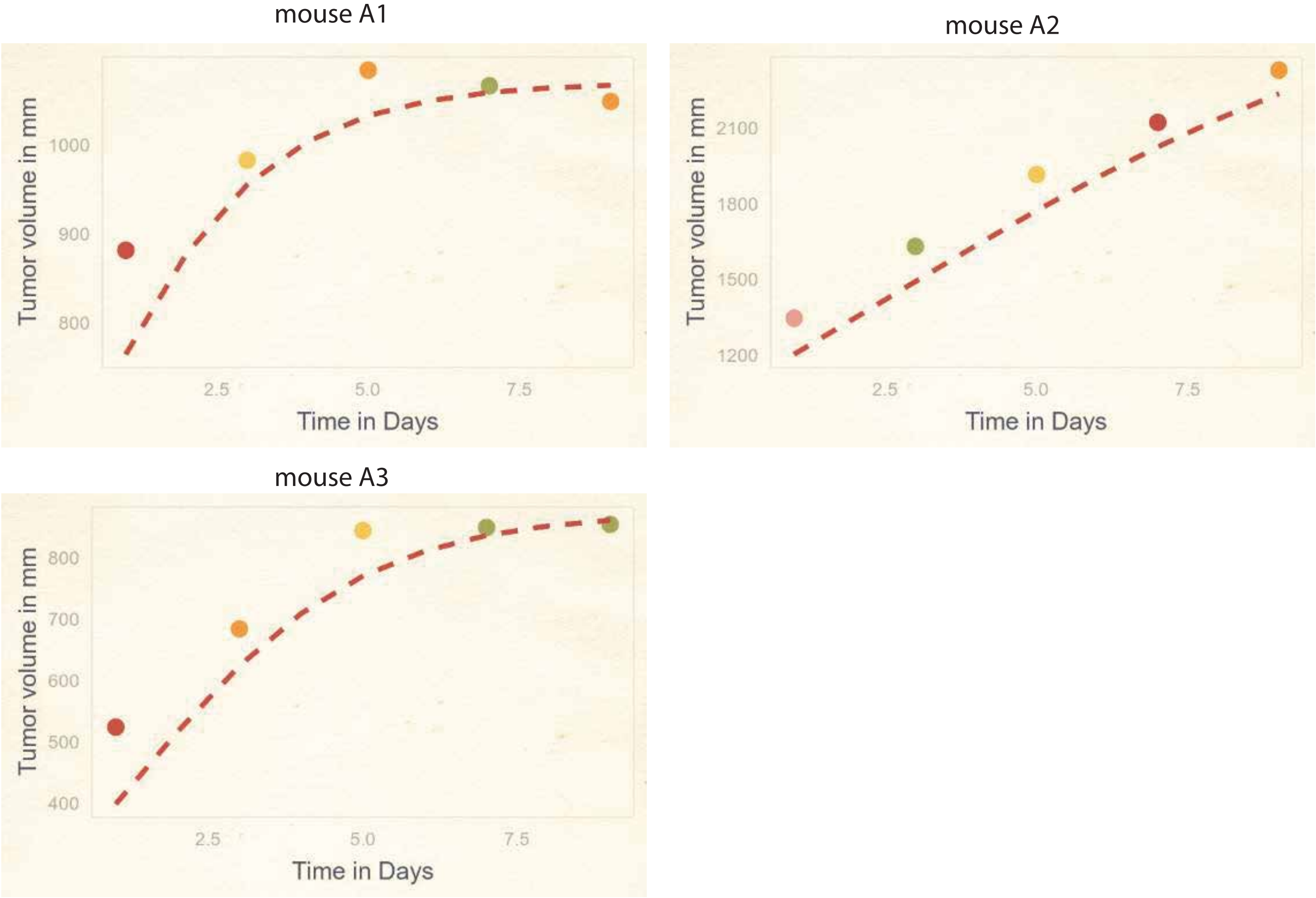

Supplementary figure 6. Neuroblastoma PDX (Hansson et. al., replicate 2)

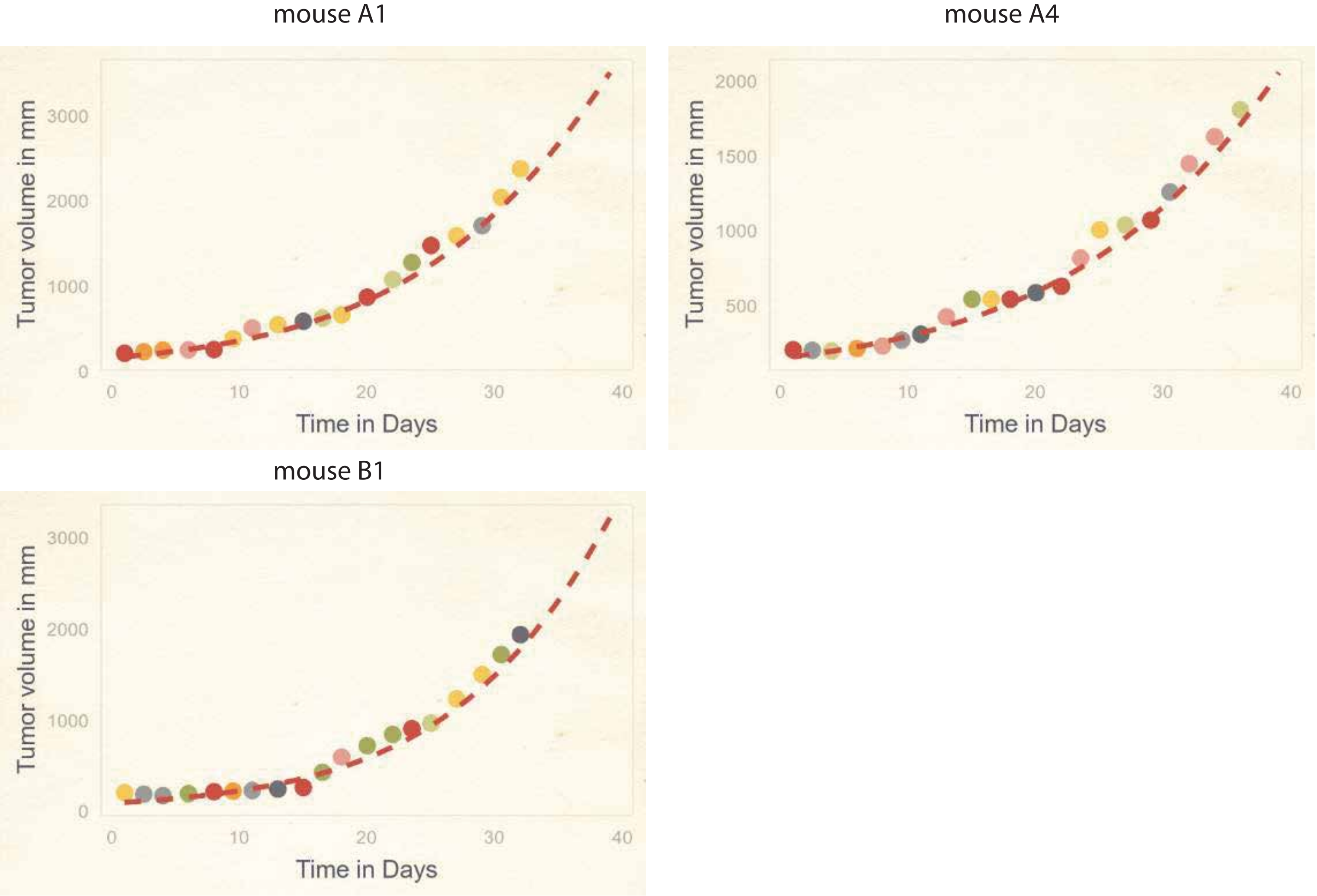

Supplementary figure 7. Neuroblastoma PDX (Radke et. al.)

mouse A2

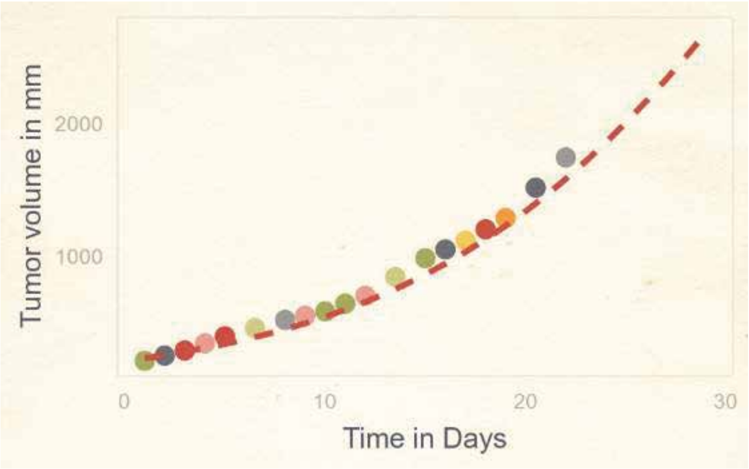

mouse A4

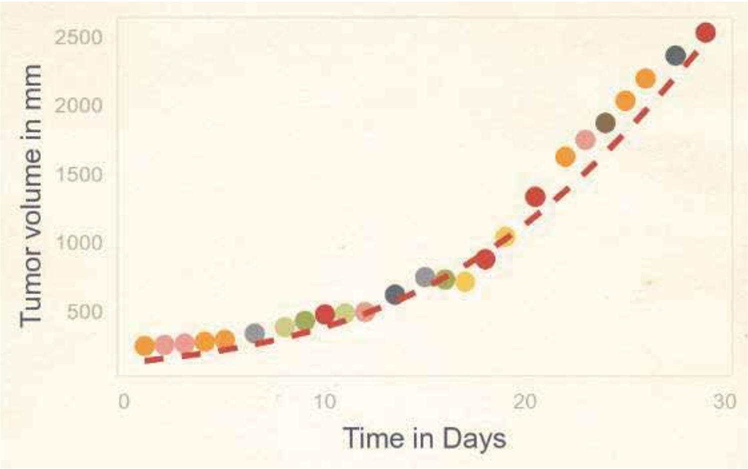

mouse A7

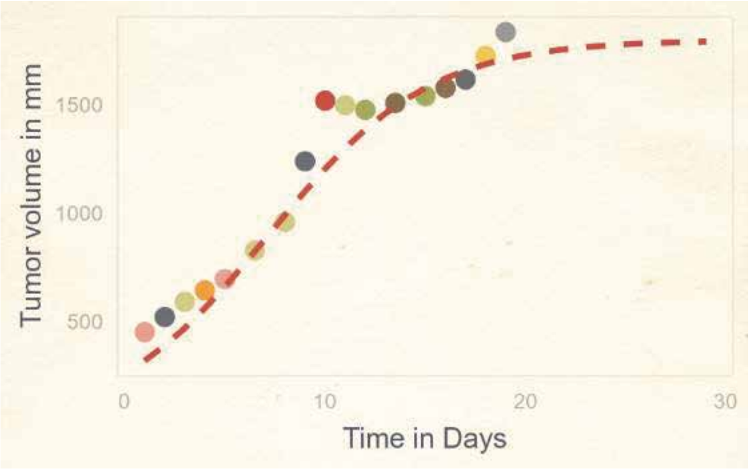

Supplementary figure 8. Neuroblastoma PDX (Mohlin et. al., replicate 1)

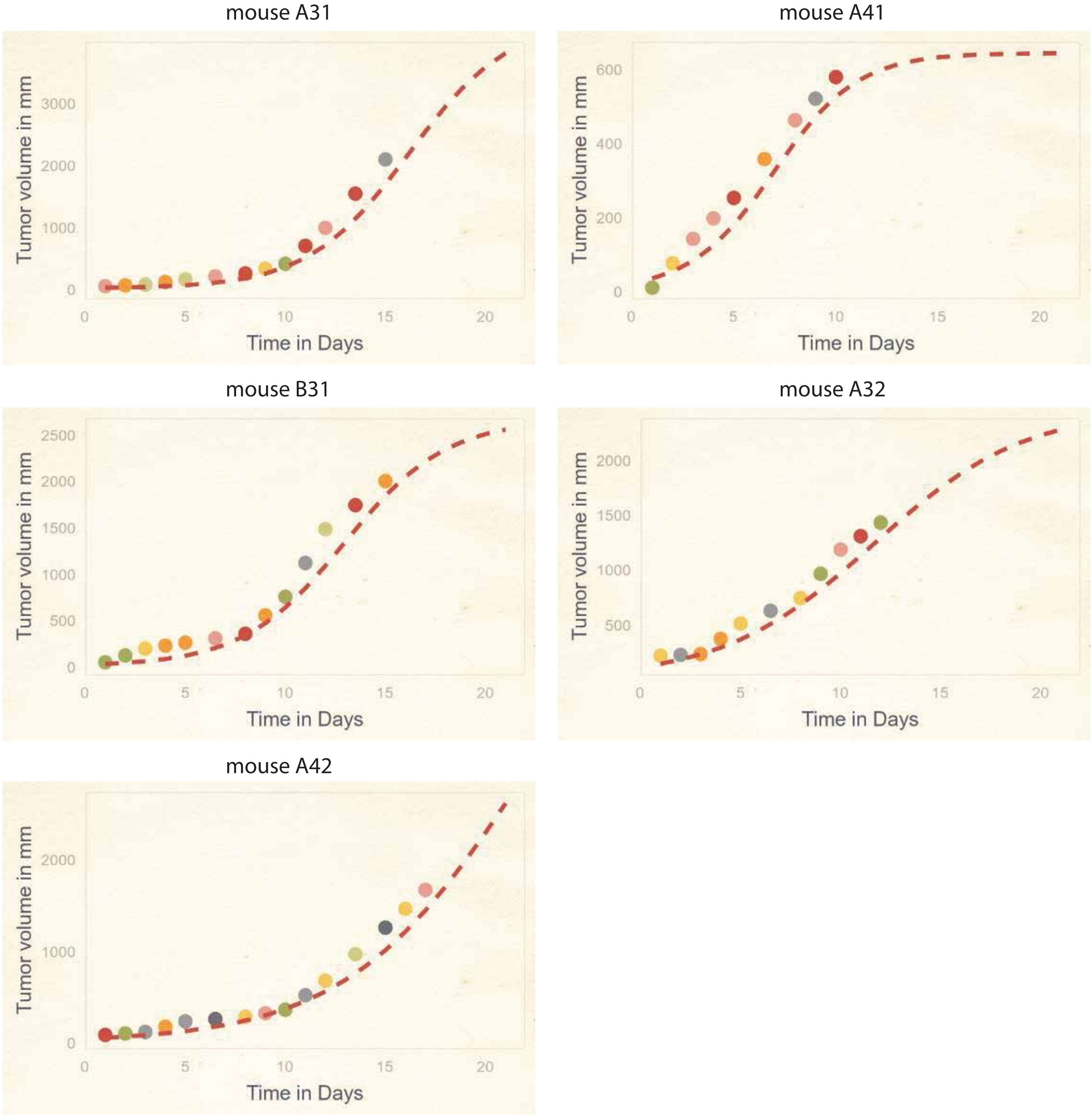

Supplementary figure 9. Neuroblastoma PDX (Mohlin et. al., replicate 2)

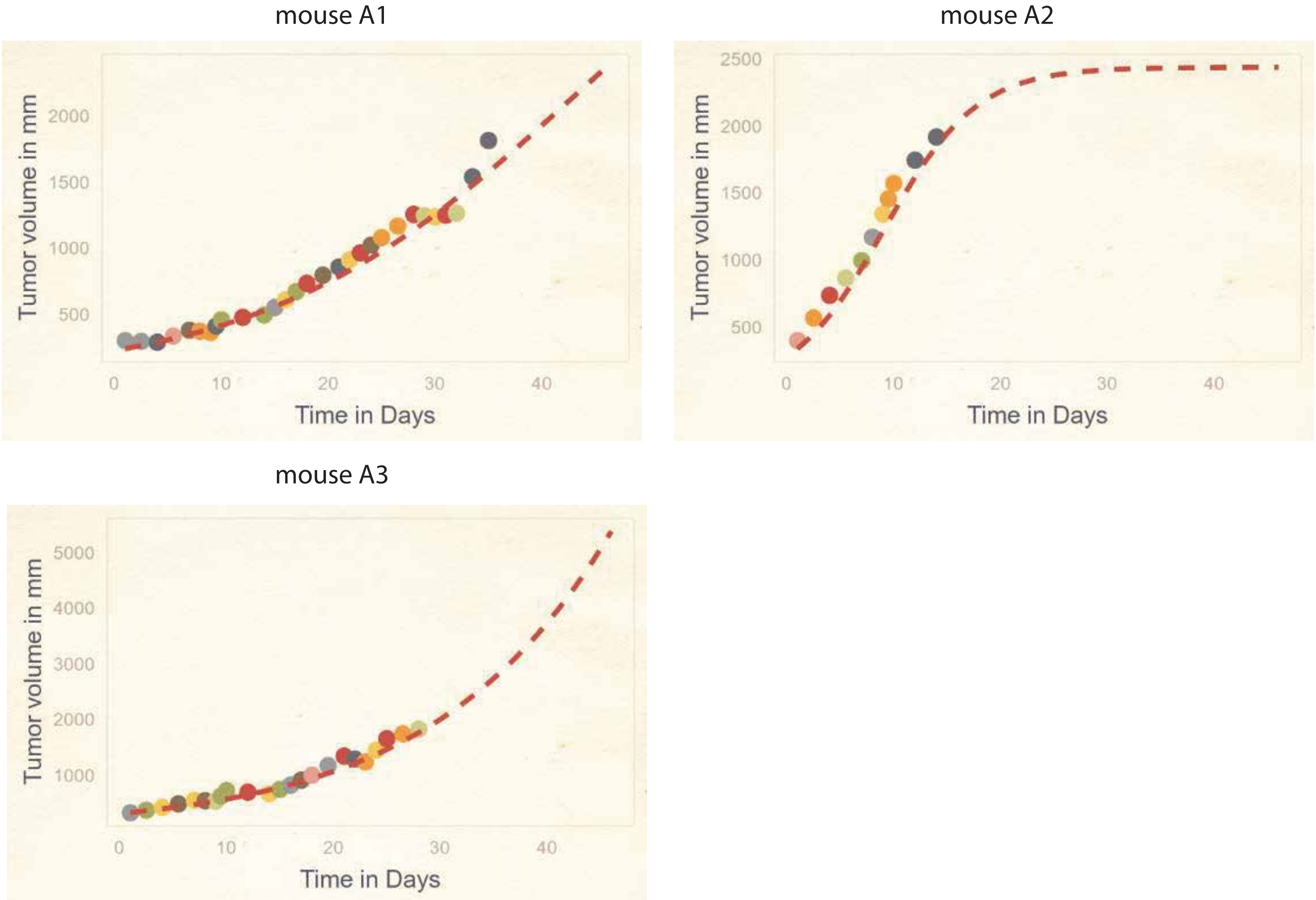

Supplementary figure 10. Neuroblastoma PDX (Mohlin et. al., replicate 3)

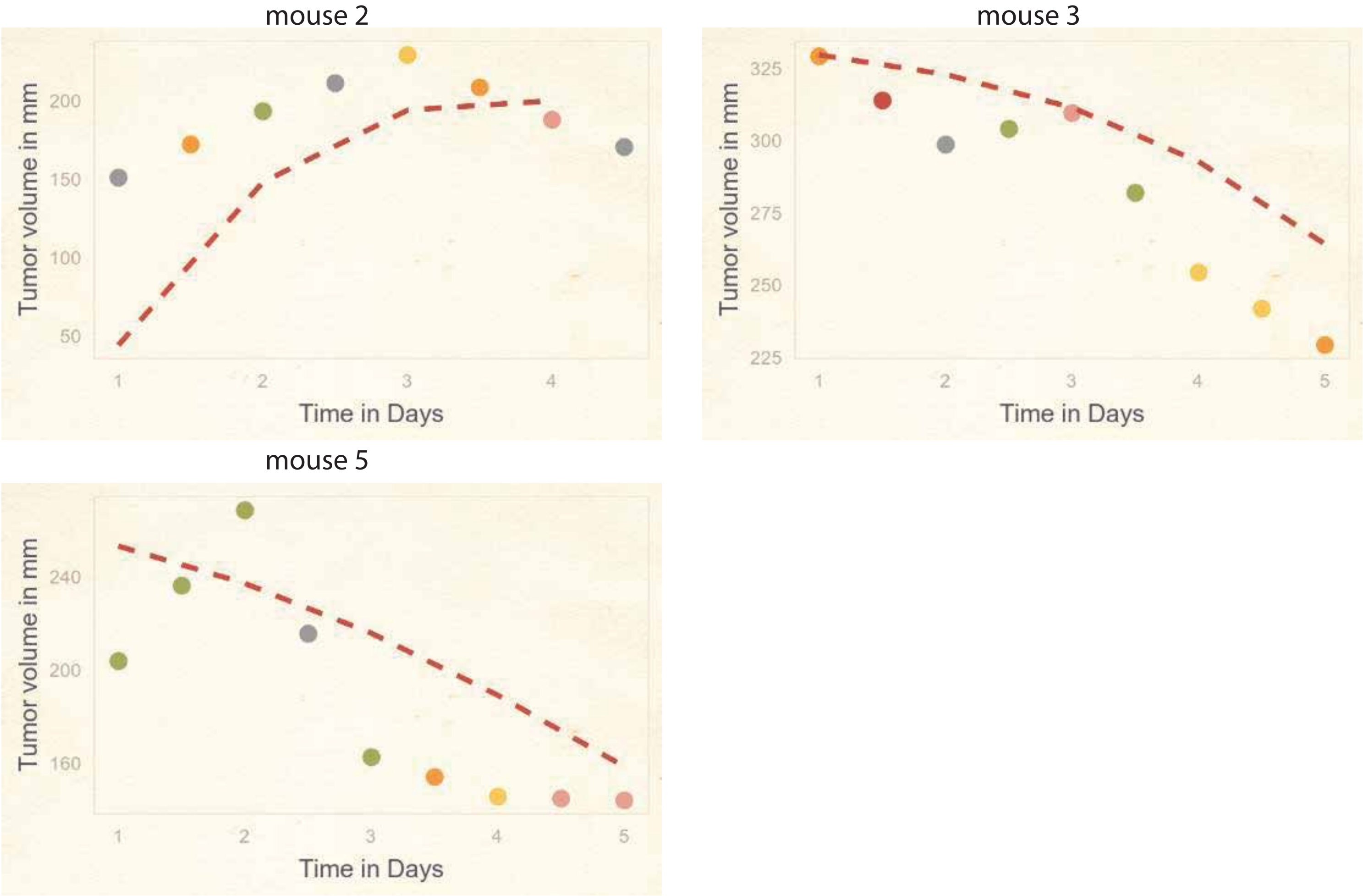

Supplementary figure 11. Neuroblastoma PDX (Mohlin et. al., replicate 4)

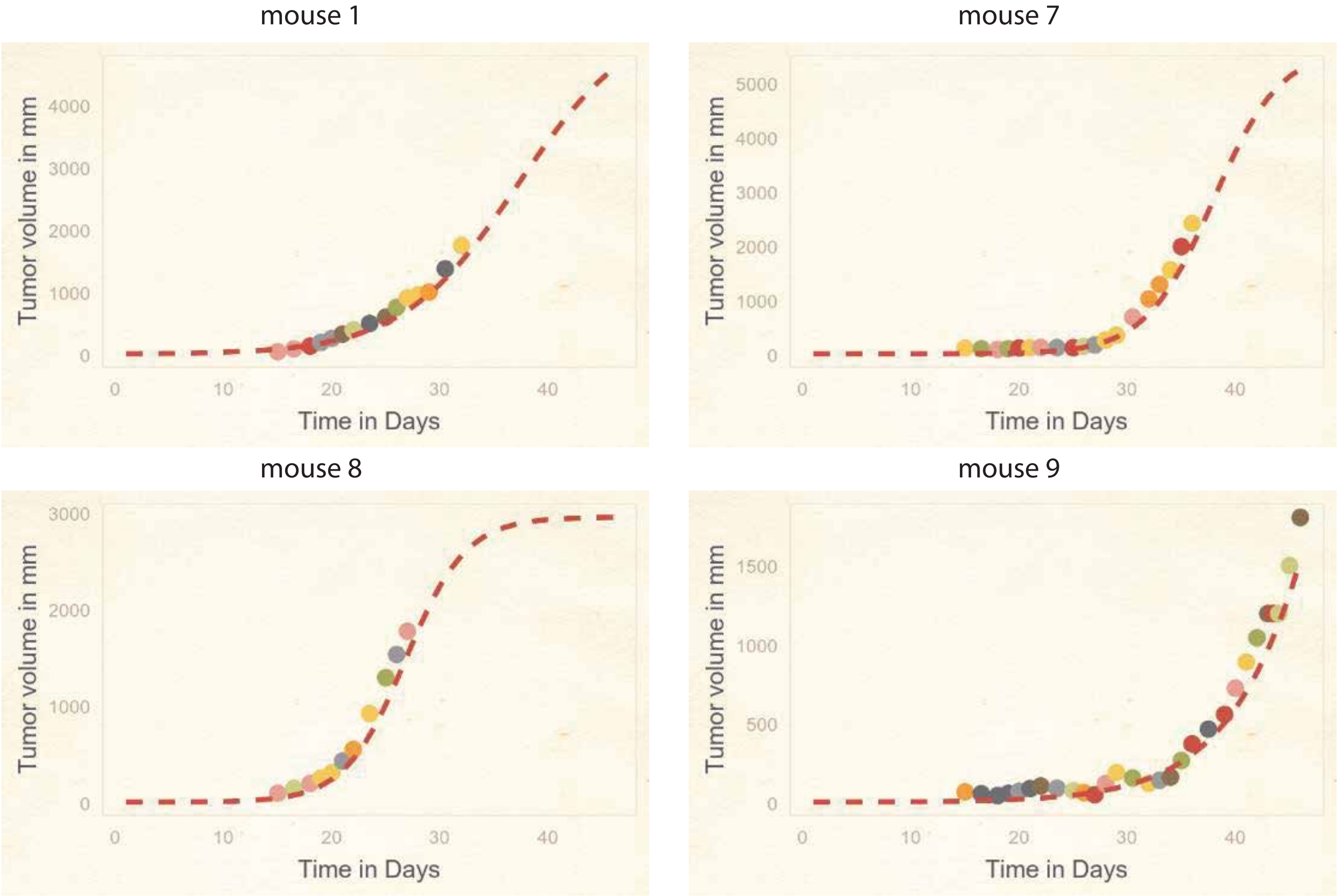

Supplementary figure 12. Neuroblastoma tumor initiating cells (Mohlin et. al.)

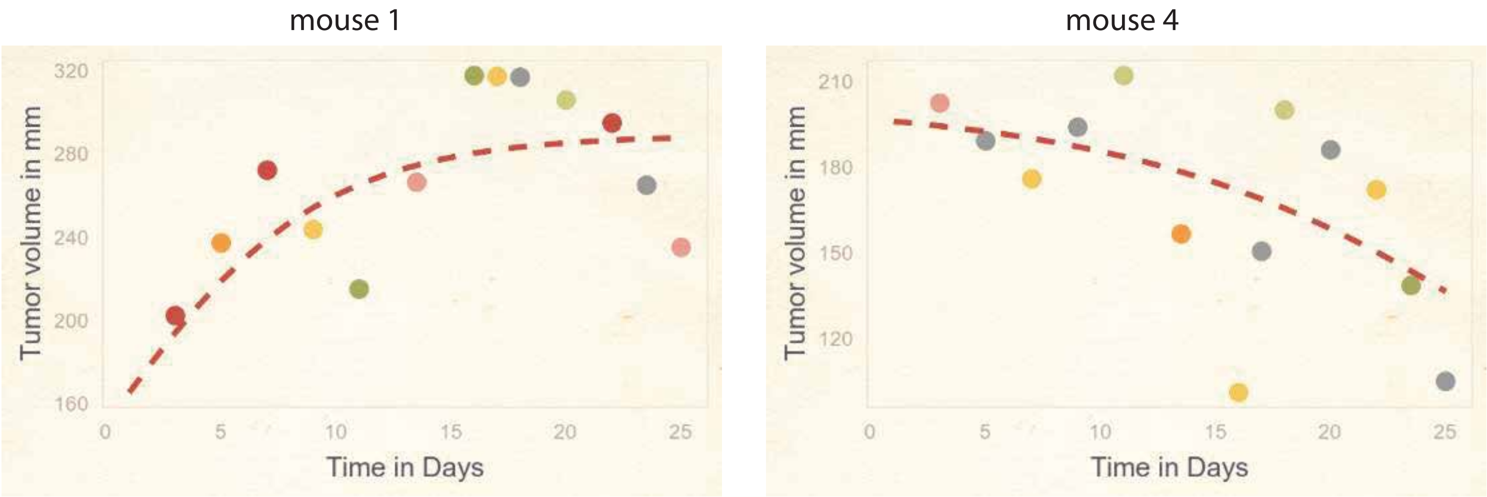

Supplementary figure 13. Neuroblastoma PDX (Manas et. al., *PDX1*, replicate 1)

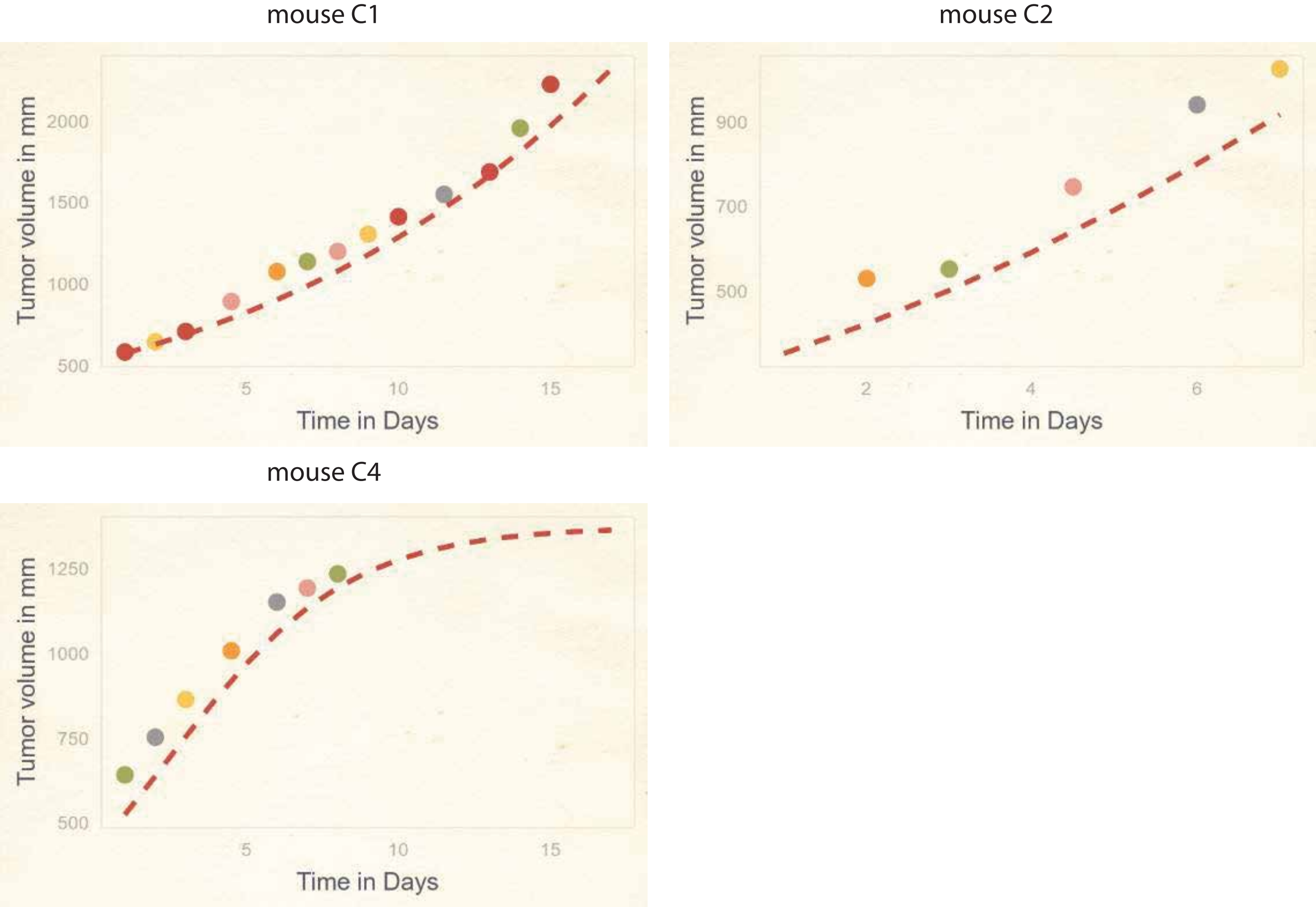

Supplementary figure 14. Neuroblastoma PDX (Manas et. al., *PDX1*, replicate 2)

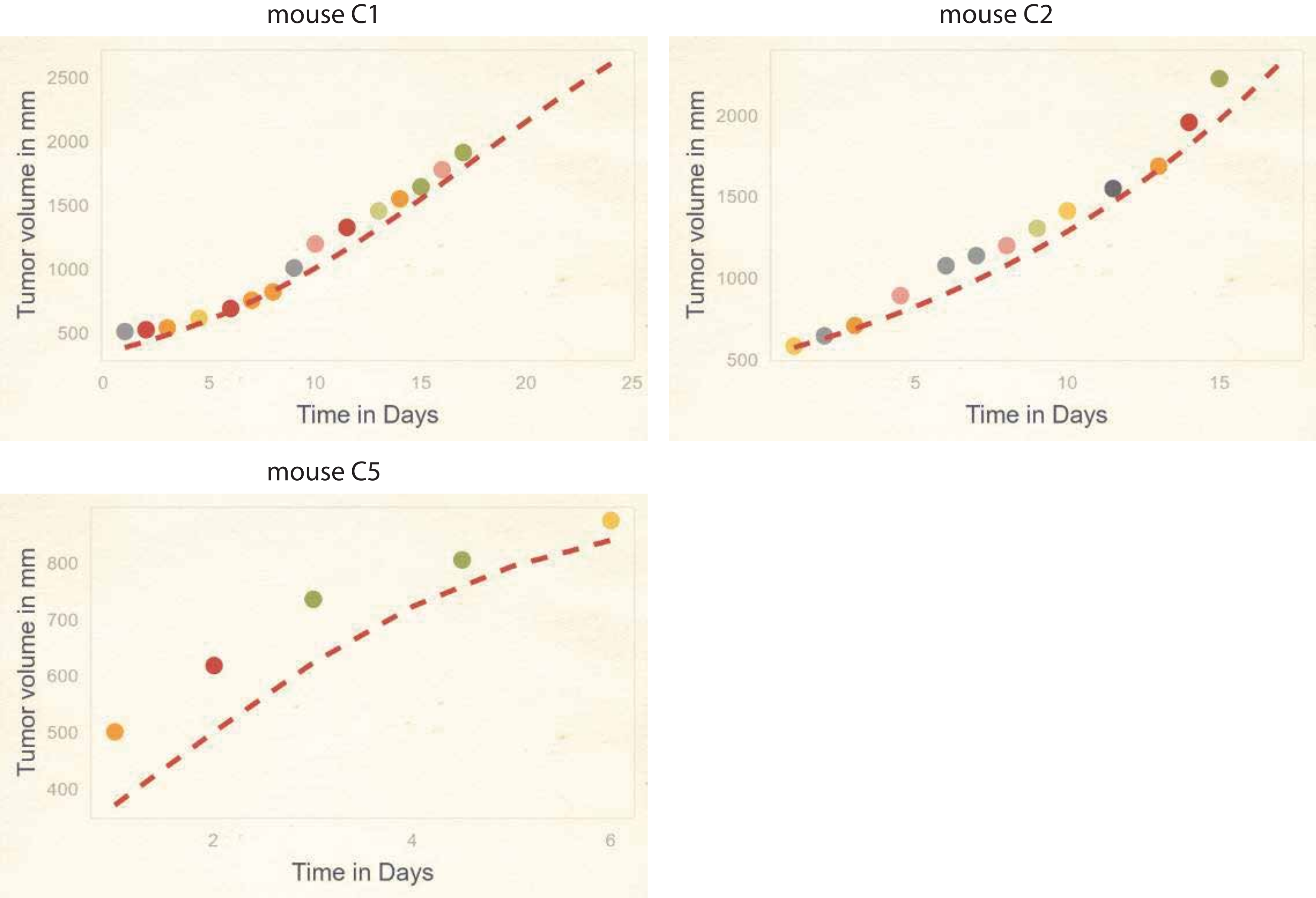

Supplementary figure 15. Neuroblastoma PDX (Manas et. al., *PDX2*)

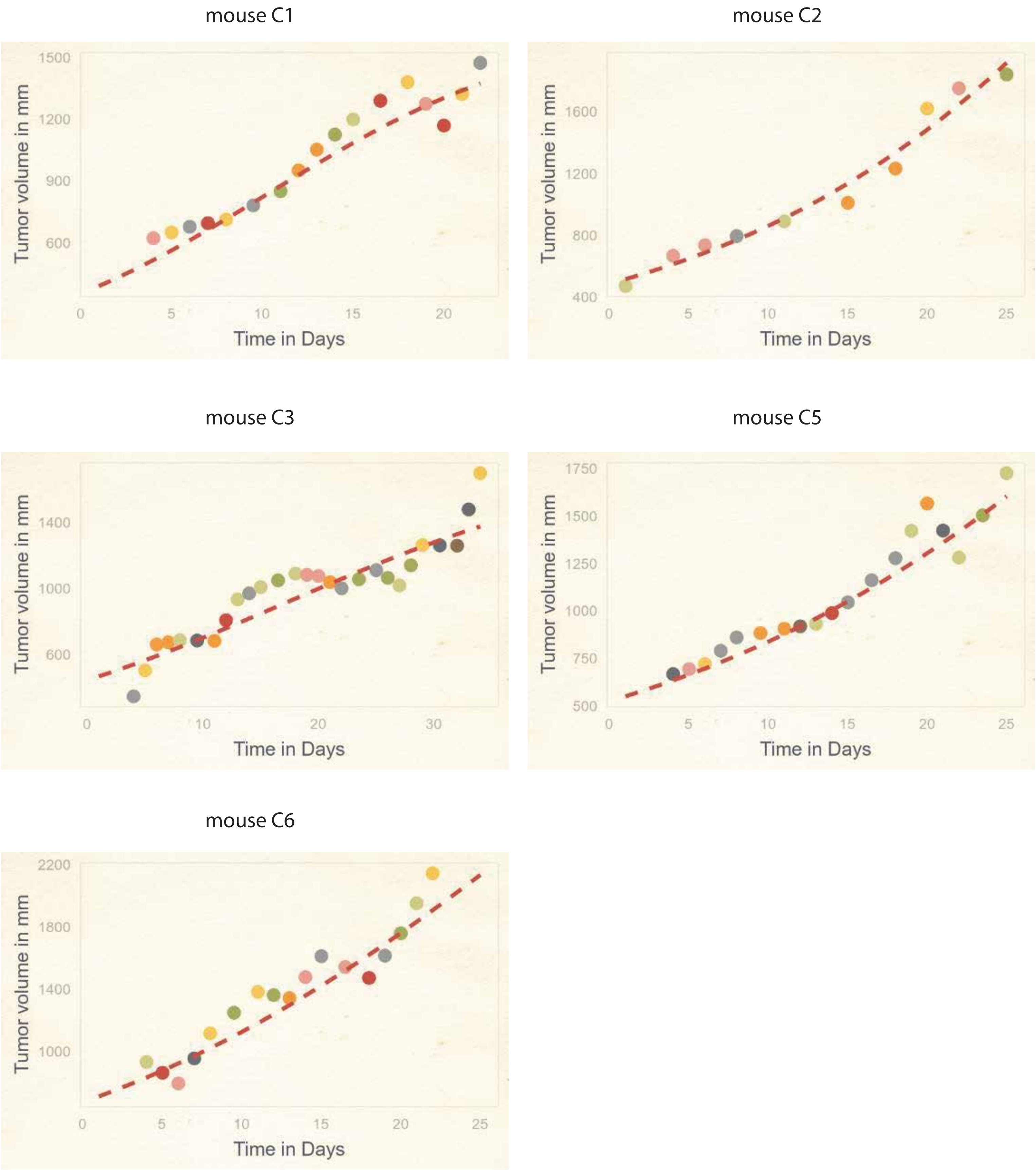

Supplementary figure 16. Neuroblastoma PDX (Manas et. al., *PDX3*, replicate 1)

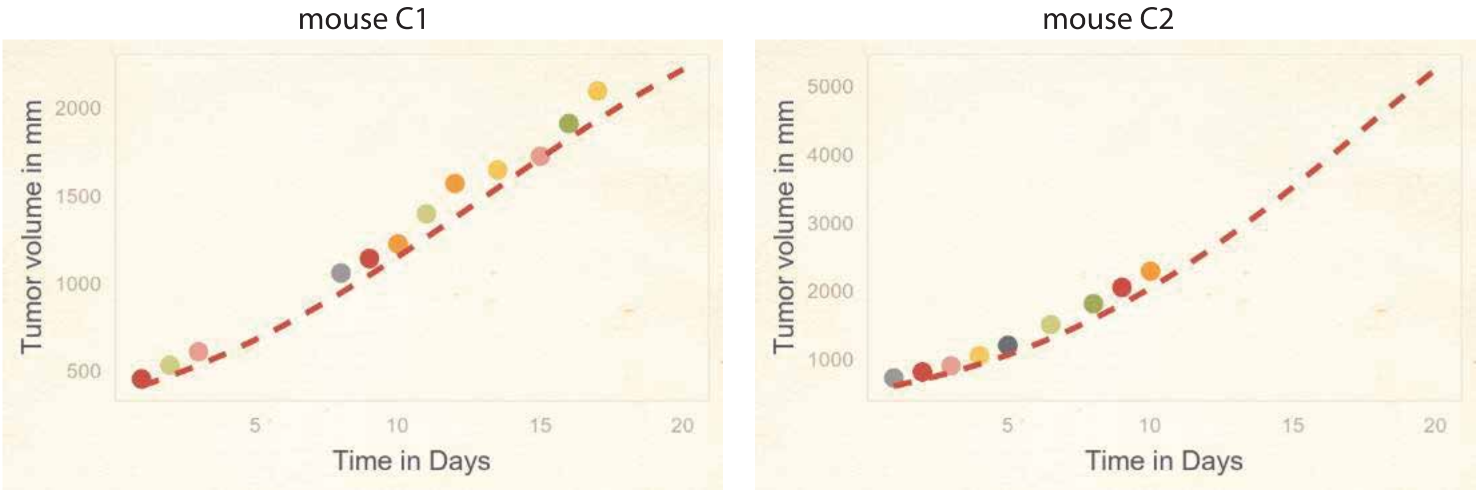

Supplementary figure 17. Neuroblastoma PDX (Manas et. al., *PDX3*, replicate 2)

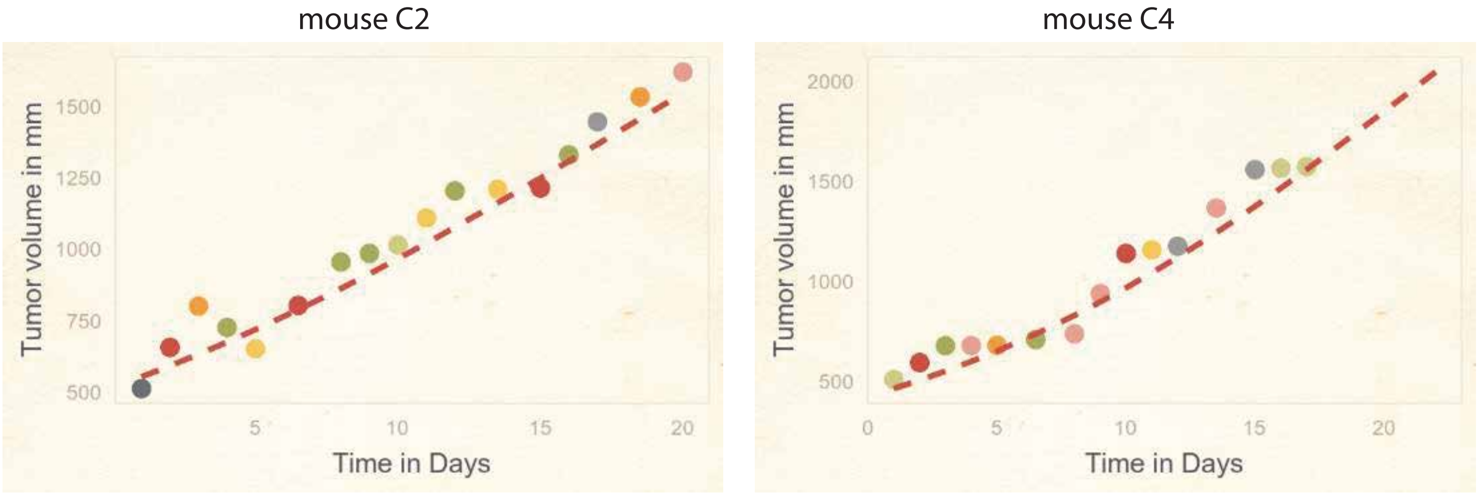

Supplementary figure 18. Neuroblastoma PDX (Manas et. al., *PDX3*, replicate 3)

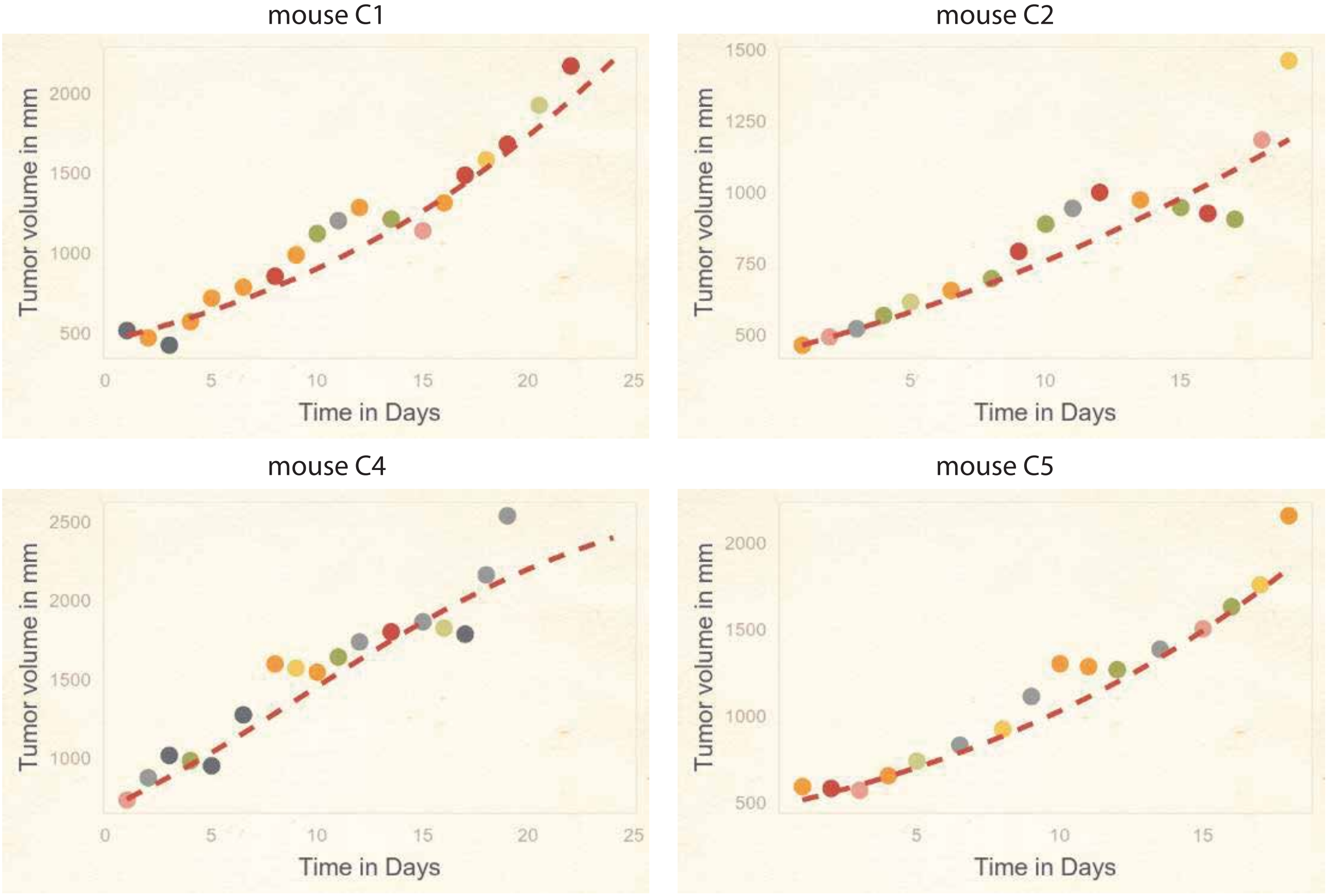

Supplementary figure 19. Wilms tumor PDX KT47

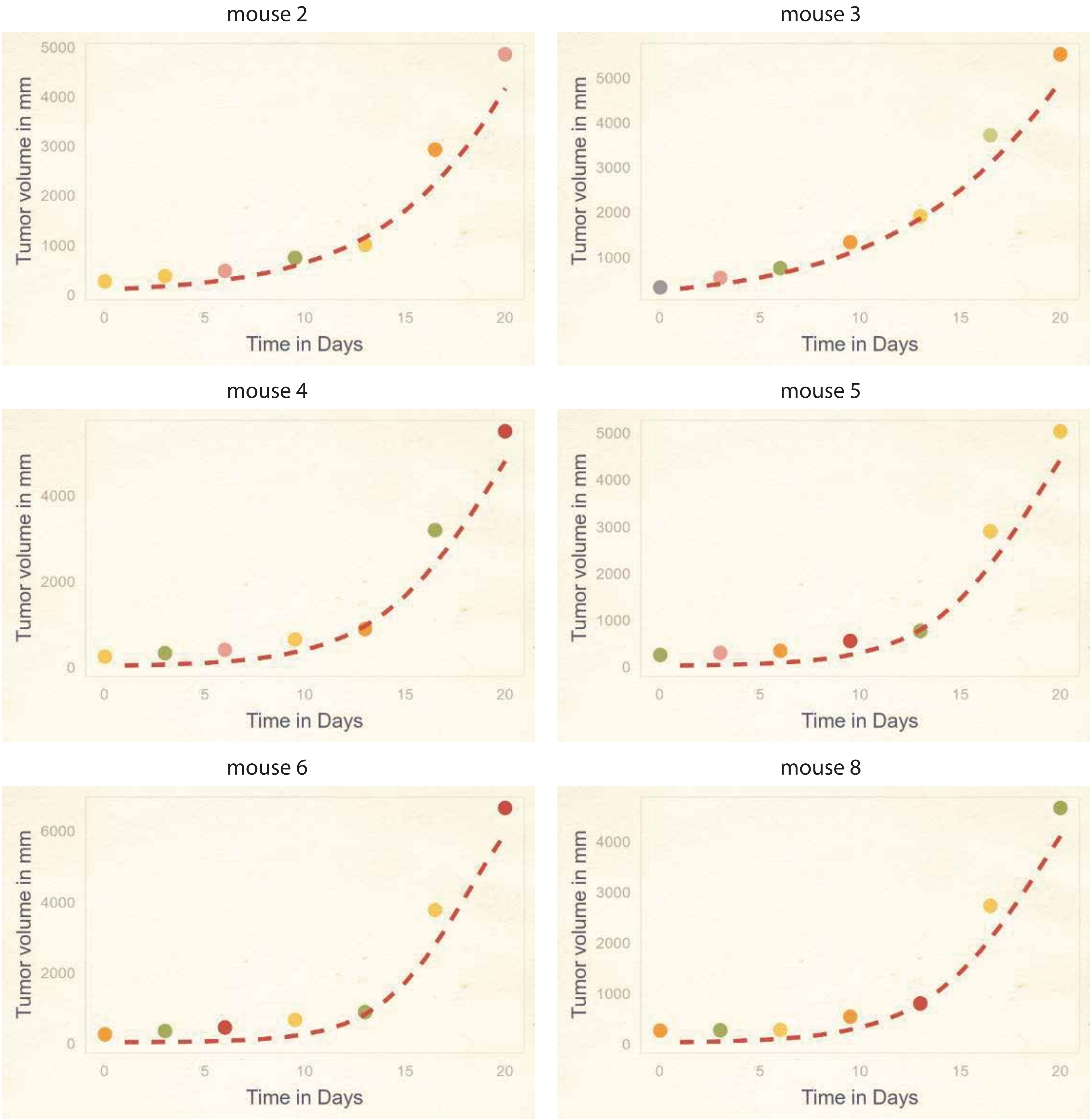

Supplementary figure 20. Wilms tumor PDX KT53

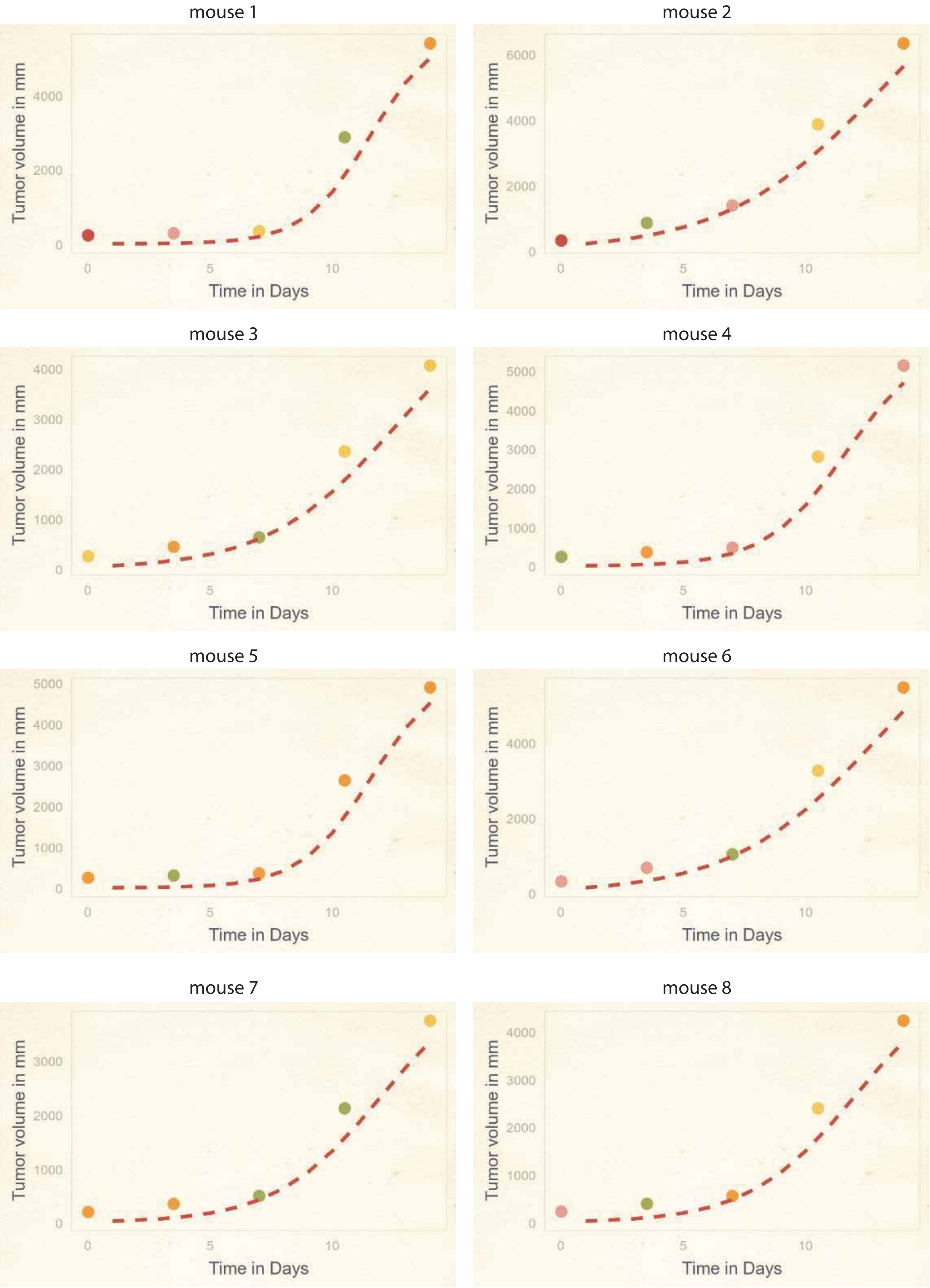

Supplementary figure 21. Wilms tumor PDX KT51

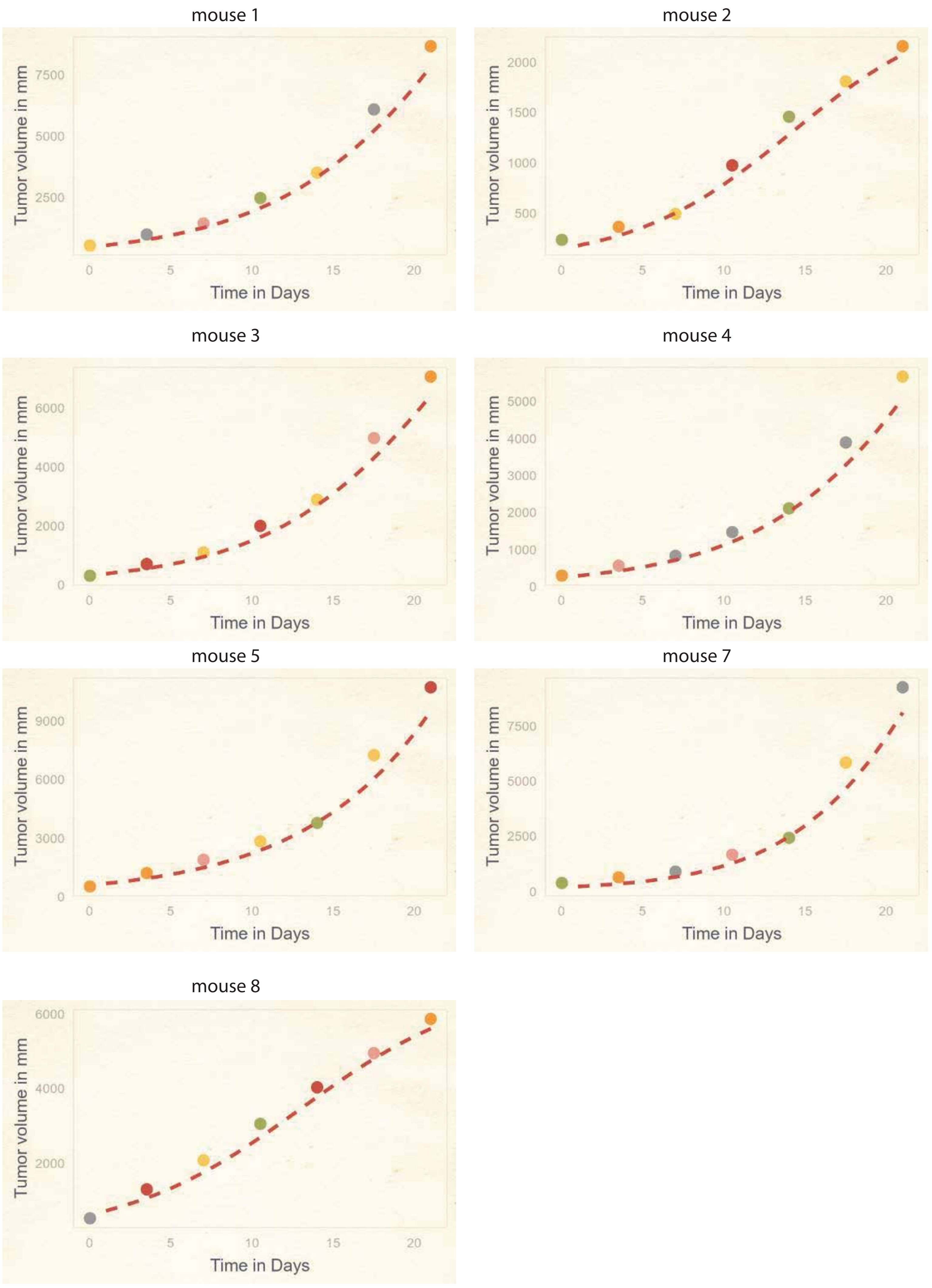

Supplementary figure 22. Wilms tumor PDX KT75

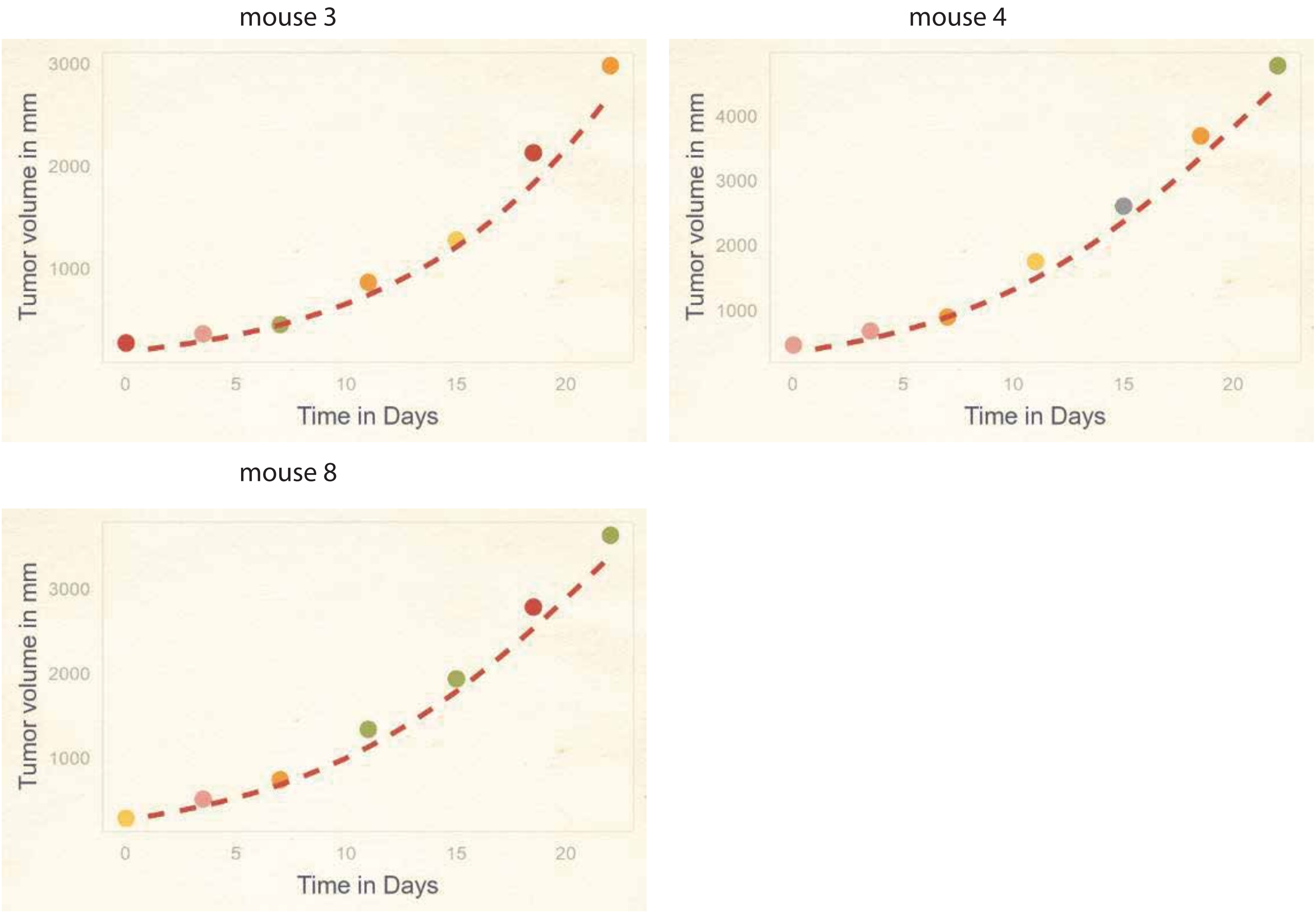

Supplementary figure 23. Wilms tumor PDX KT43

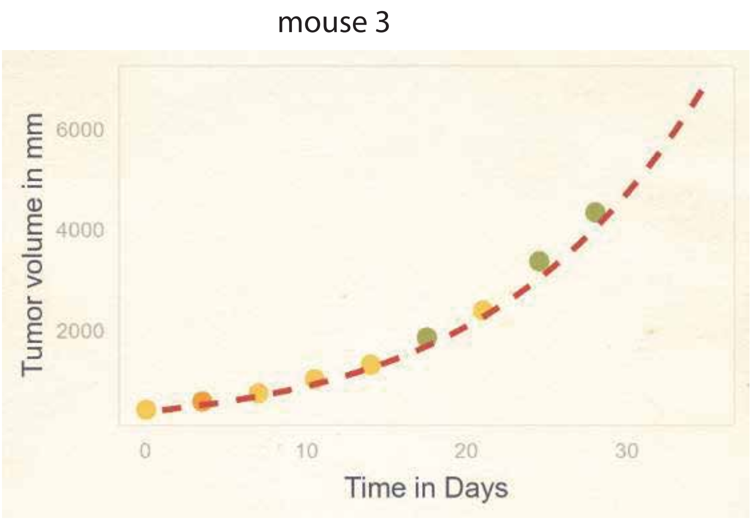

Supplementary figure 24. A549 lung cancer cell line (replicate 1)

mouse 1

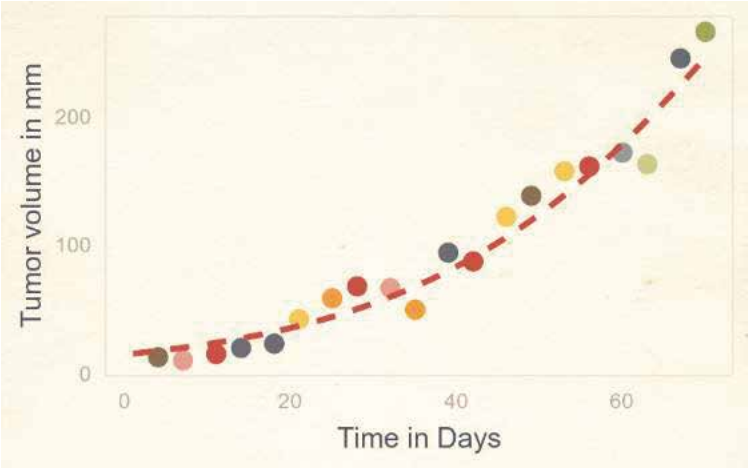

mouse 2

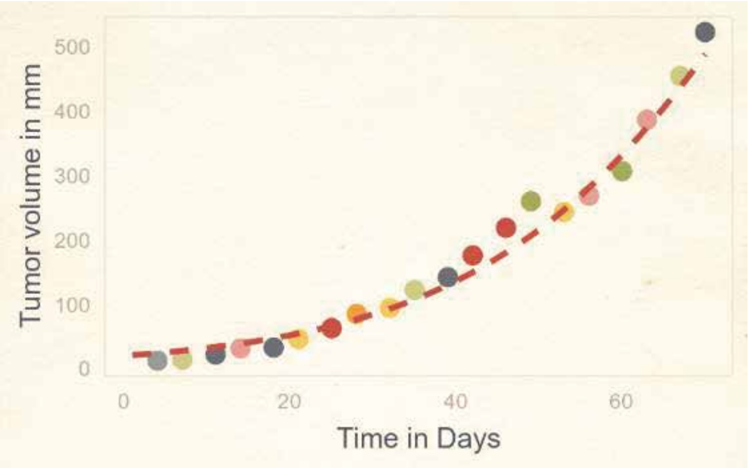

mouse 4

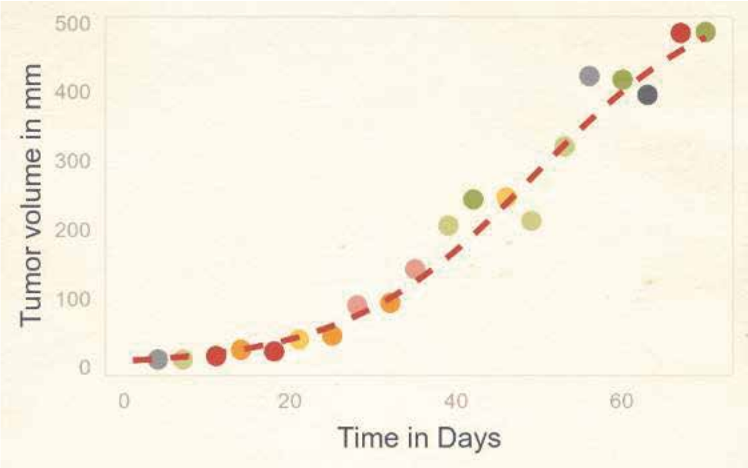

mouse 5

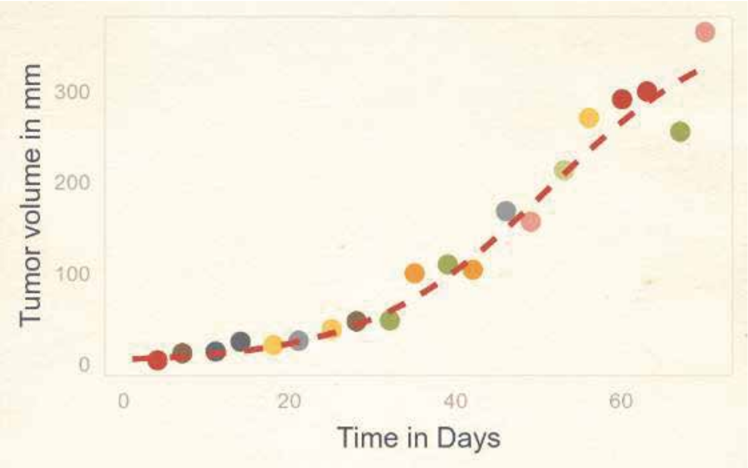

mouse 6

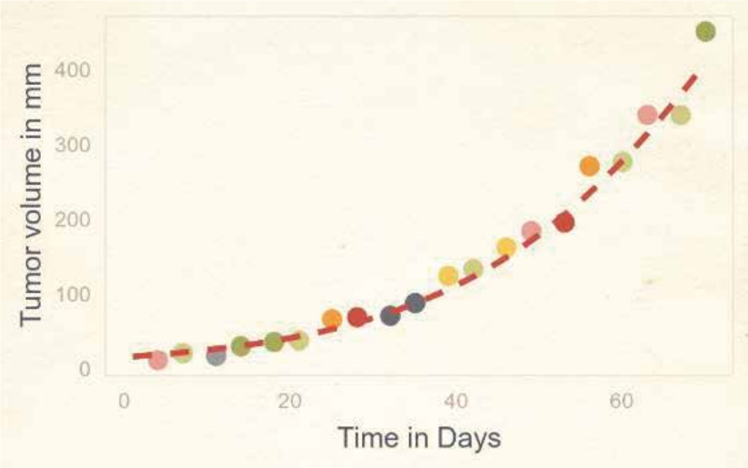

mouse 7

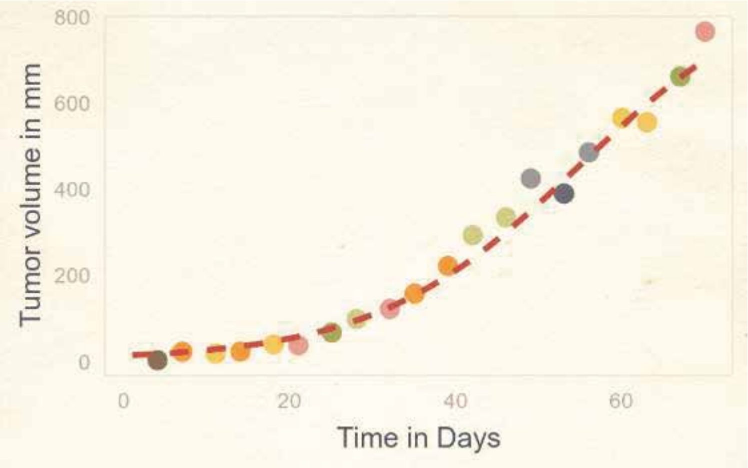

mouse 7

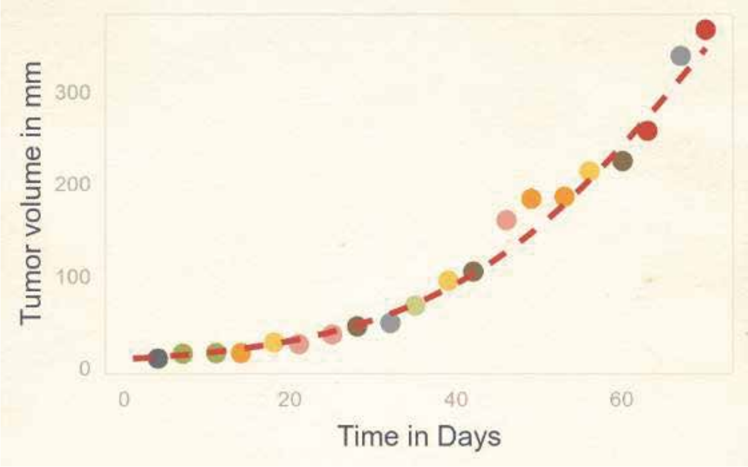

Supplementary figure 25. A549 lung cancer cell line (replicate 2)

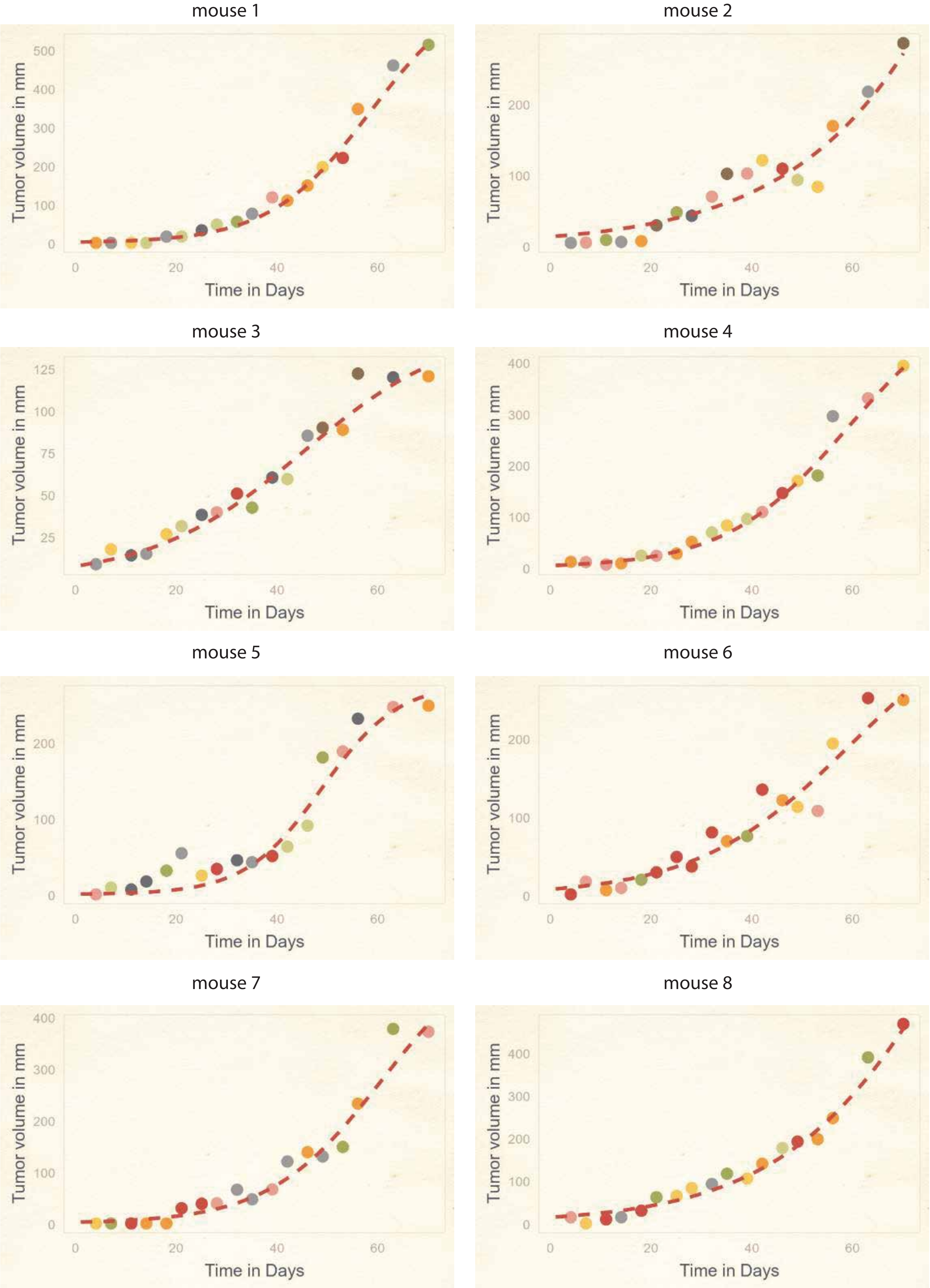

Supplementary figure 26. H441 lung cancer cell line

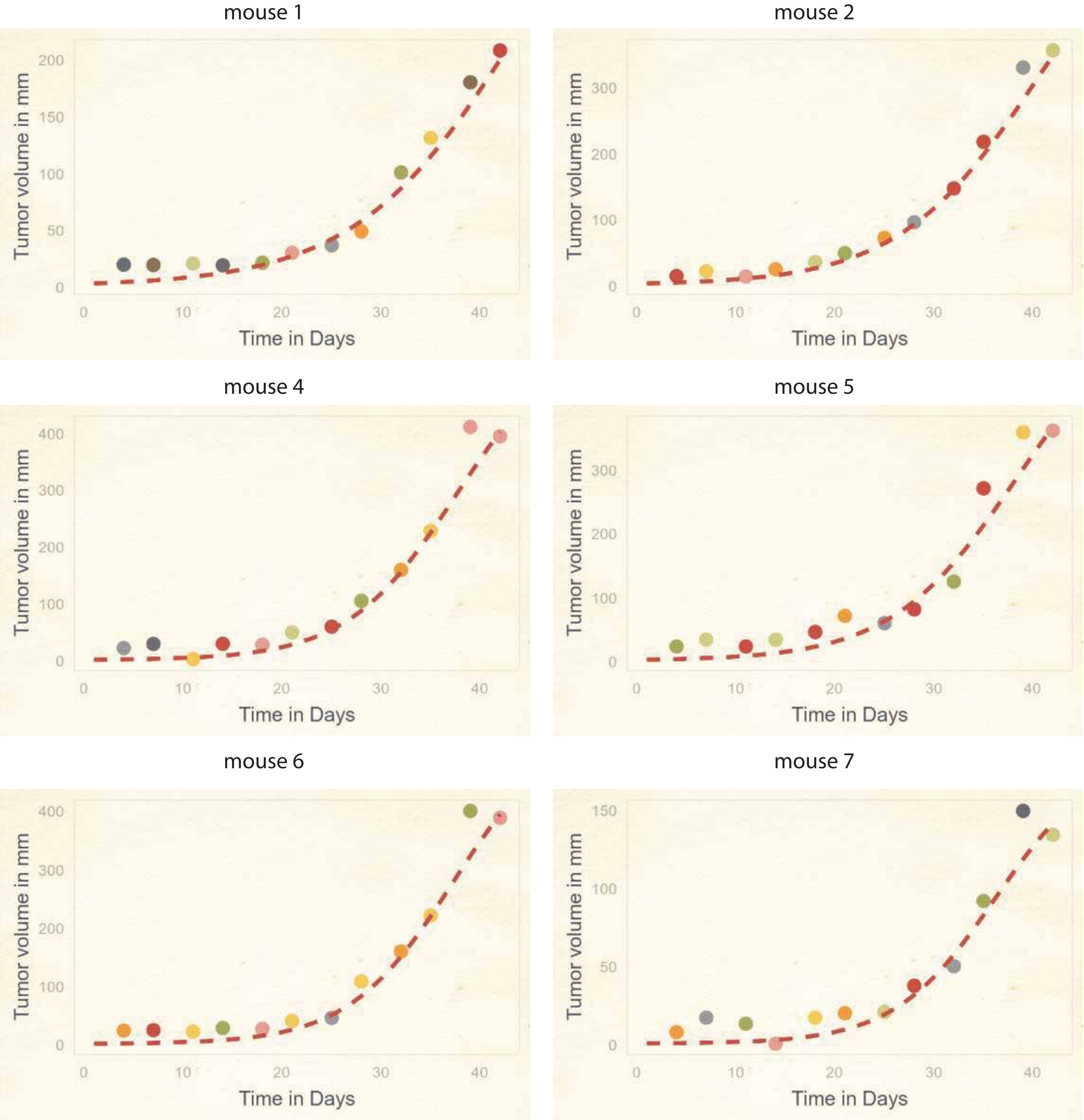

Supplementary figure 27. H520 lung cancer cell line (replicate 1)

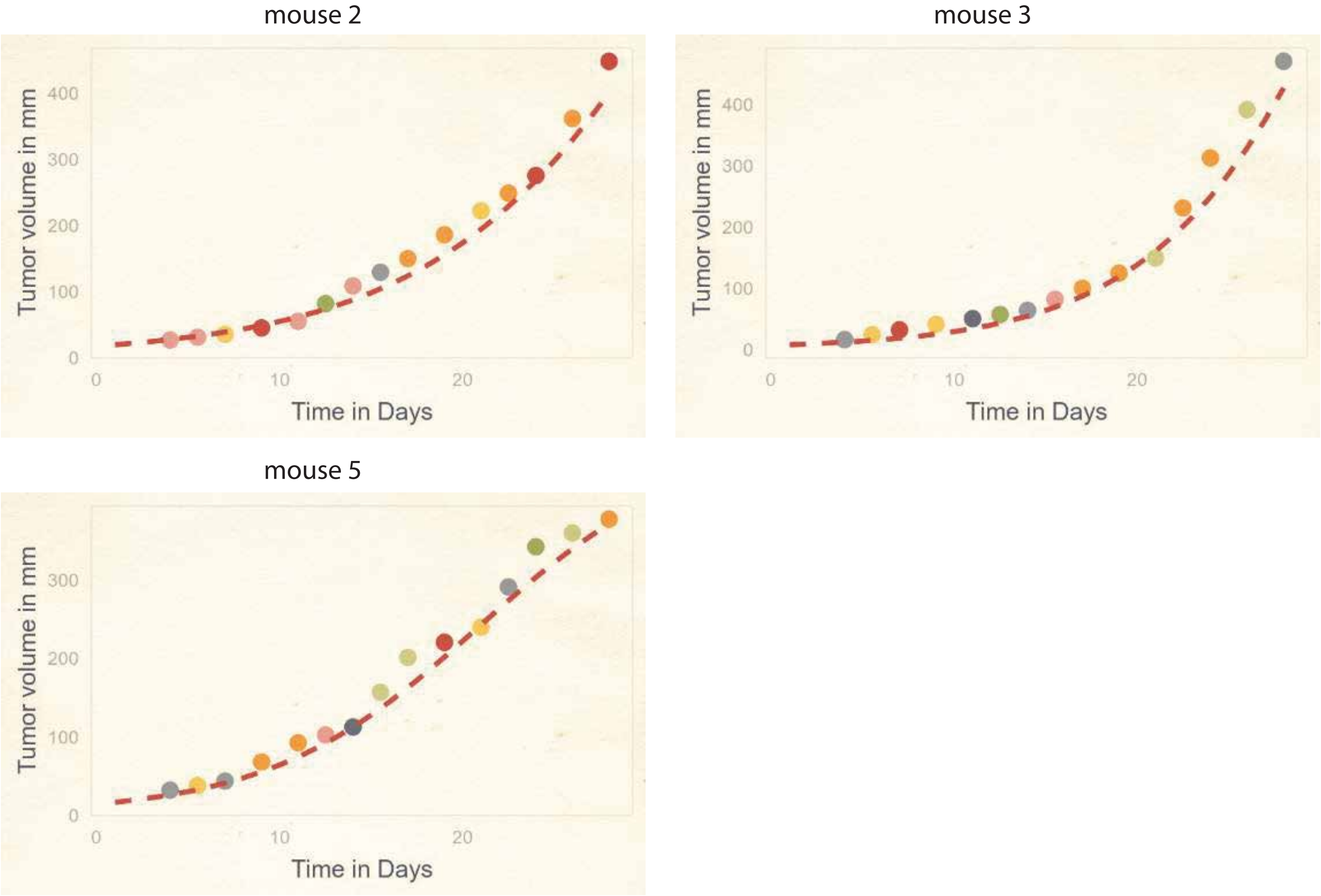

Supplementary figure 28. H520 lung cancer cell line (replicate 2)

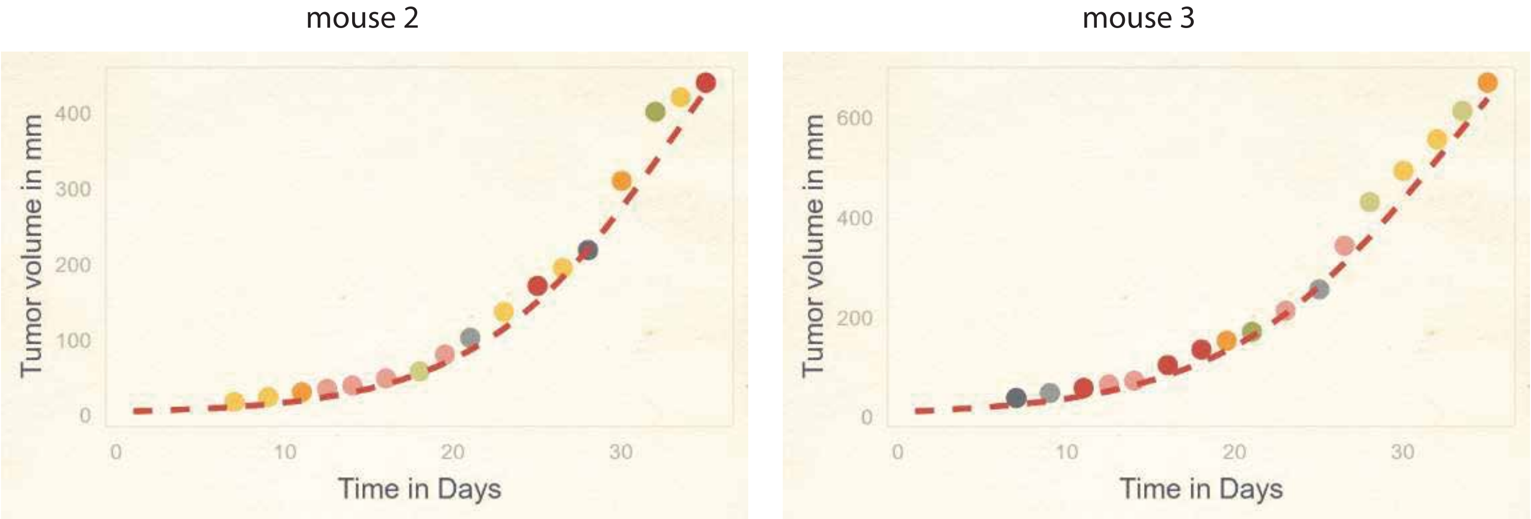

Supplementary figure 29. MCF7 breast cancer cell line

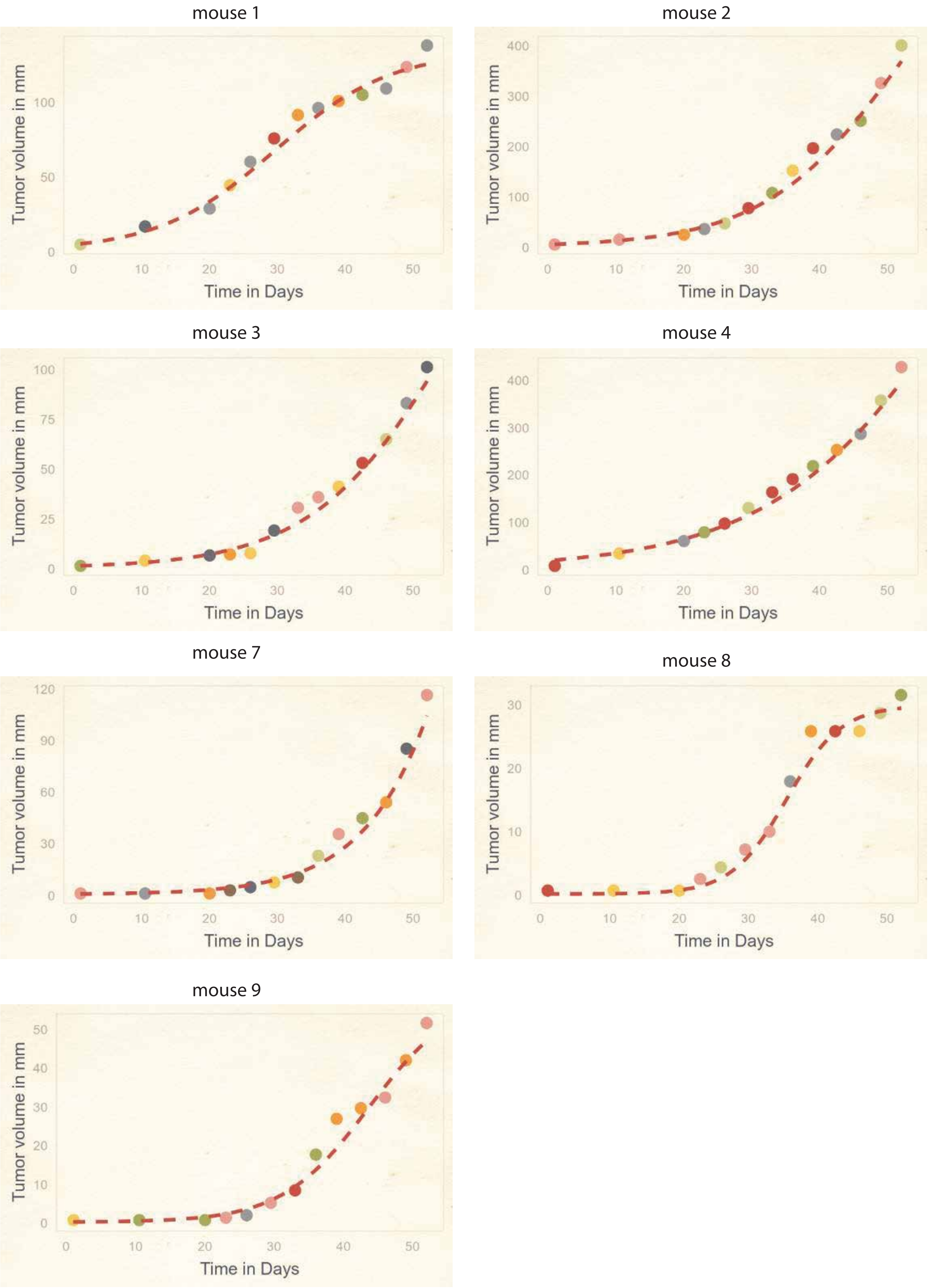

Supplementary figure 30. MDA-MB-231 breast cancer cell line

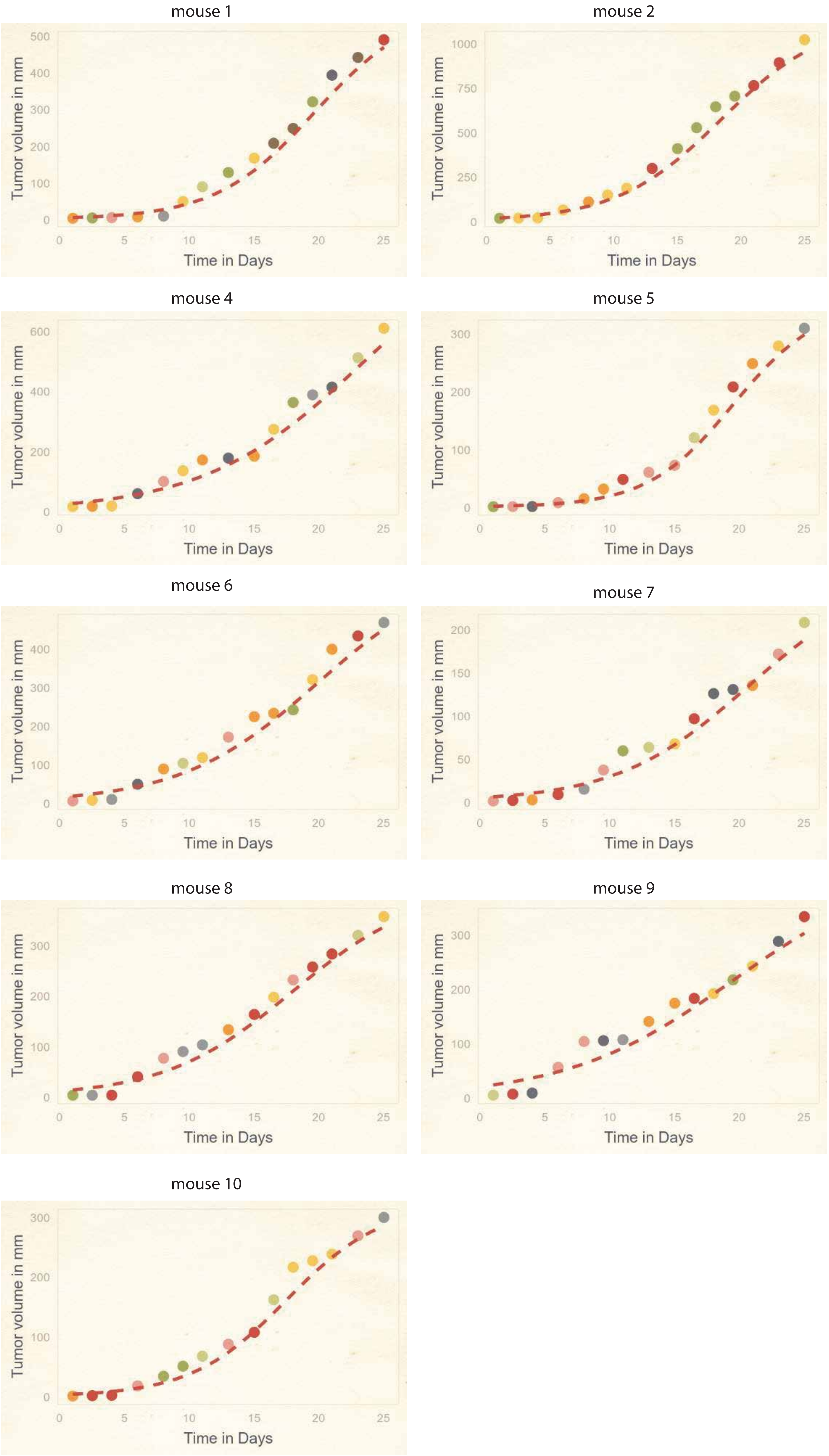

Supplementary Figure 31

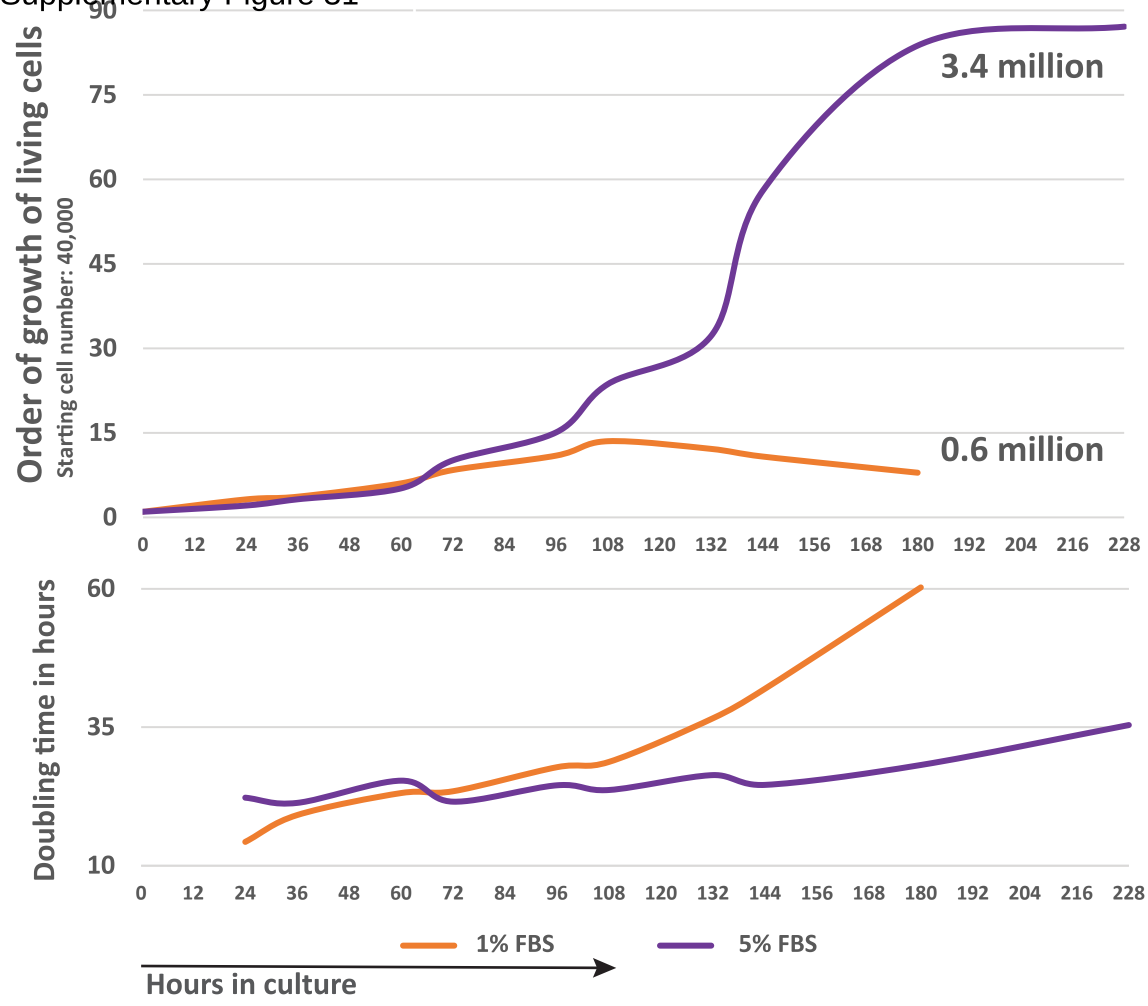

Supplementary Figure 32

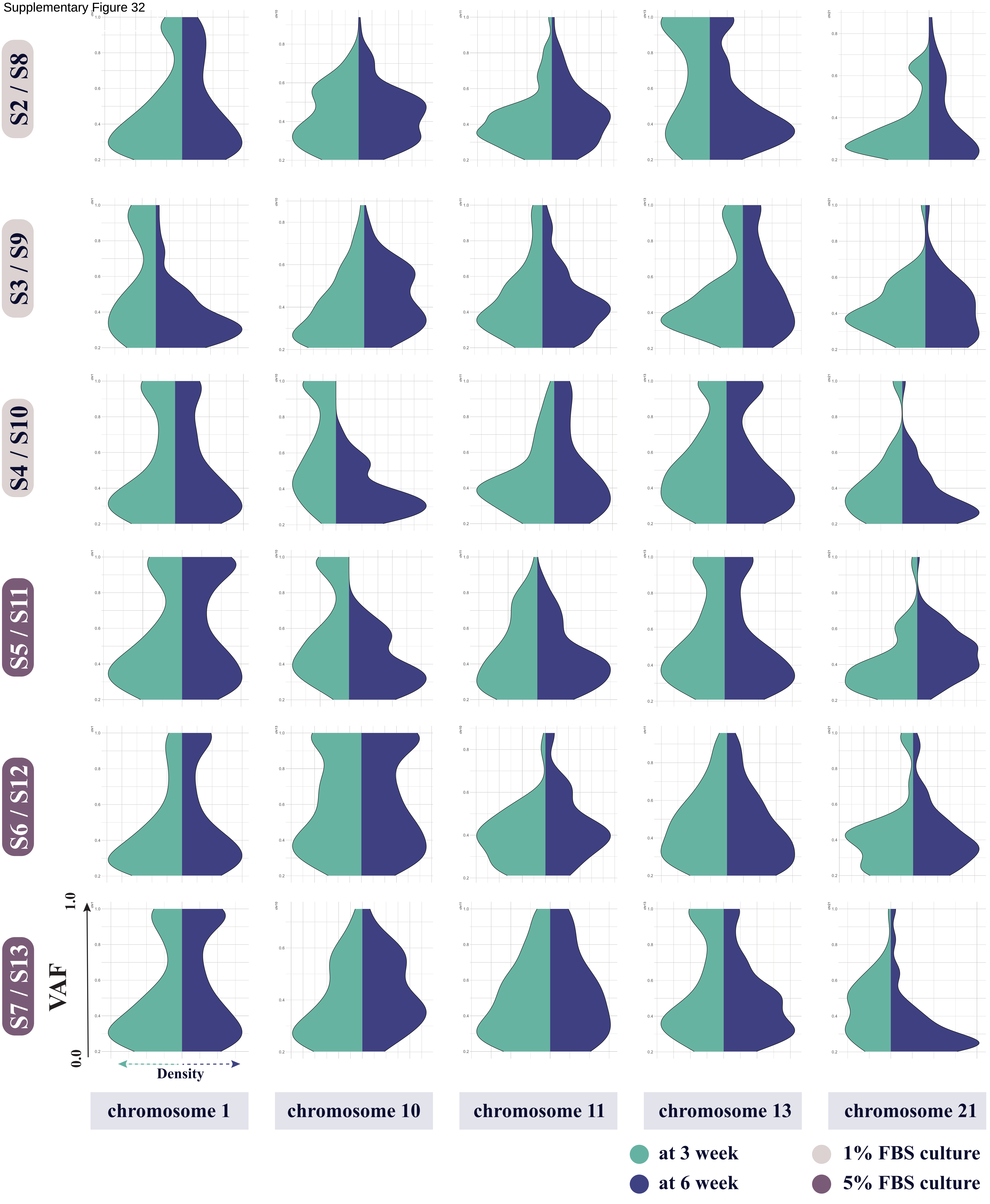

Supplementary Figure 33

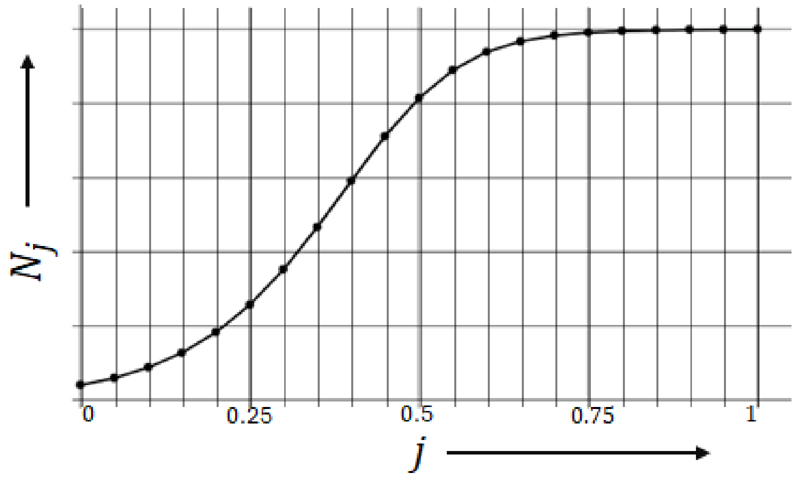

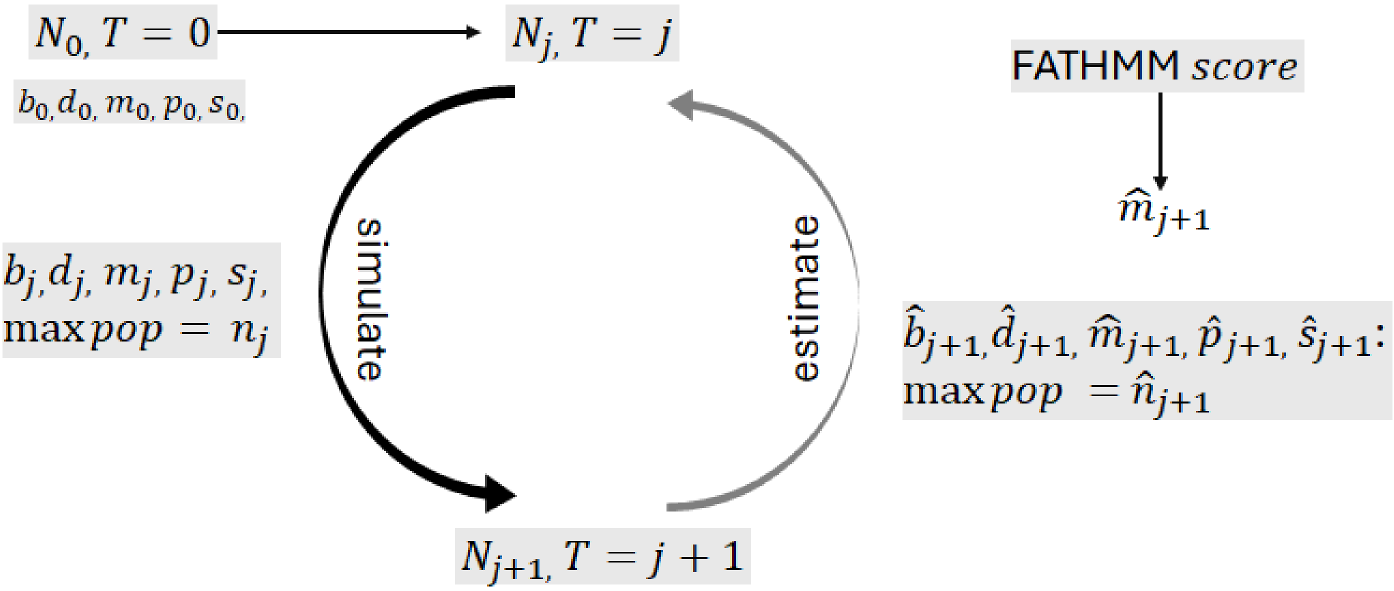

Supplement: Supplementary file 1 — Supplementary Information. [file 41598_2025_7407_MOESM1_ESM.pdf]
